# IGF2BP1, a Conserved Regulator of RNA Turnover in Cancer

**DOI:** 10.3389/fmolb.2021.632219

**Published:** 2021-03-22

**Authors:** Markus Glaß, Danny Misiak, Nadine Bley, Simon Müller, Sven Hagemann, Bianca Busch, Alexander Rausch, Stefan Hüttelmaier

**Affiliations:** Institute of Molecular Medicine, Martin Luther University Halle-Wittenberg, Halle, Germany

**Keywords:** IGF2BP1, cancer, E2F, AURKA, HDLBP, YWHAZ

## Abstract

The oncofetal IGF2 mRNA-binding protein 1 (IGF2BP1) promotes tumor progression in a variety of solid tumors and its expression is associated with adverse prognosis. The main role proposed for IGF2BP1 in cancer cells is the stabilization of mRNAs encoding pro-oncogenic factors. Several IGF2BP1-RNA association studies, however, revealed a plethora of putative IGF2BP1-RNA targets. Thus, at present the main conserved target RNAs and pathways controlled by IGF2BP1 in cancer remain elusive. In this study, we present a set of genes and cancer hallmark pathways showing a conserved pattern of deregulation in dependence of IGF2BP1 expression in cancer cell lines. By the integrative analysis of these findings with publicly available cancer transcriptome and IGF2BP1-RNA association data, we compiled a set of prime candidate target mRNAs. These analyses confirm a pivotal role of IGF2BP1 in controlling cancer cell cycle progression and reveal novel cancer hallmark pathways influenced by IGF2BP1. For three novel target mRNAs identified by these studies, namely AURKA, HDLBP and YWHAZ, we confirm IGF2BP1 mRNA stabilization. In sum our findings confirm and expand previous findings on the pivotal role of IGF2BP1 in promoting oncogenic gene expression by stabilizing target mRNAs in a mainly 3’UTR, m^6^A-, miRNA-, and potentially AU-rich element dependent manner.

## 1 Introduction

The oncofetal IGF2 mRNA-binding protein 1 (IGF2BP1) is a crucial regulator of tumor and stem cell fate and its elevated expression in a multitude of tumors is associated with poor prognosis (Degrauwe et al., 2016; Hattori et al., 2016). Despite the conserved regulation of cancer cell properties like proliferation, migration, invasion and metastasis, conserved effector pathways and target RNAs of IGF2BP1 in cancer remain largely elusive (Stöhr et al., 2012; Hamilton et al., 2013; Gutschner et al., 2014; Busch et al., 2016; Müller et al., 2019; Rosenfeld et al., 2019; Müller et al., 2020). In previous studies, IGF2BP1 was shown to promote mRNA stability in distinct solid cancer models and stem cells, reviewed in: Bell et al. (2013), Cao et al. (2018), Huang X. et al. (2018). A common theme of this regulation is the control of mRNA turnover by impairing microRNA-dependent downregulation of target mRNAs encoding pro-oncogenic factors (Müller et al., 2018). This was originally described for the IGF2BP1-dependent enhancement of BTRC (β-TrCP1) expression (Noubissi et al., 2006; Elcheva et al., 2009). In support of these findings the impairment of miRNA-dependent regulation was demonstrated in a variety of cancer cell models for various mRNAs including pro-oncogenic factors like LIN28B and HMGA2 (Busch et al., 2016), MITF (Goswami et al., 2015), MKI67 (Gutschner et al., 2014; Müller et al., 2020) and SRF (Müller et al., 2019). However, only for the regulation of E2F-driven gene expression, a strongly conserved role of IGF2BP1 in promoting gene expression of E2F1-3 transcription factors and some of the target transcripts, e.g., MKI67, was reported (Müller et al., 2020). In support of these findings, studies in distinct cancer models confirm a role of IGF2BP1 in promoting additional cancer hallmark pathways, including MYC/MYCN-driven gene expression demonstrated in ovarian cancer (Köbel et al., 2007), liver cancer (Gutschner et al., 2014; Huang H. et al., 2018) and neuroblastoma (Bell et al., 2015), as well as KRAS-driven signaling in lung adenocarcinoma (Rosenfeld et al., 2019). The post-transcriptional enhancement of pro-oncogenic gene expression by IGF2BP1 essentially relies on its four C-terminal KH (HNRNPK homology) domains, essential for RNA-association (Wegrowski et al., 1990; Farina et al., 2003). Their inactivation by point mutation of the central GxxG motif essentially abolishes the RNA-binding dependent regulation of target mRNA stabilization (Wegrowski et al., 1990; Müller et al., 2020). Most recently, IGF2BP1 was identified as a potent N6-methyladenosine (m^6^A) reader in cancer resulting in elevated RNA-association and consequently enforced expression of pro-oncogenic factors like MYC, SRF and E2F1 (Huang H. et al., 2018; Lan et al., 2019; Müller et al., 2019; Müller et al., 2020). Notably, recent findings indicate that m^6^A-dependent RNA-association of IGF2BP1 is further enhanced in cancer cells by an oncopeptide derived from LINC00266-1 (Zhu et al., 2020). Consistent with these findings, the impairment of IGF2BP1-RNA association by the small molecule BTYNB substantially interferes with the IGF2BP1-driven expression of pro-oncogenic factors in cancer cells and impairs the tumor growth in mouse models (Mahapatra et al., 2017; Müller et al., 2020). Moreover, the inhibition of IGF2BP1-RNA association by BTYNB proved beneficial in promoting the potency and/or efficacy of cancer therapeutics targeting IGF2BP1-driven effectors or pathways, as demonstrated for the CDK4/6 inhibitor palbociclip (Müller et al., 2020). Collectively, these findings suggest that the inhibition of IGF2BP1-dependent mRNA stabilization has therapeutic benefit on its own and may further enhance therapeutic efficacy in combined treatment strategies. RNA-association CLIP (crosslinking and immunoprecipitation) studies of IGF2BP1 in HEK293, embryonic stem cells and cancer cells suggested a variety of candidate target mRNAs of IGF2BP1, preferred 3’UTR binding and proposed short binding motifs of the protein (Hafner et al., 2010; Conway et al., 2016; Van Nostrand et al., 2016; Huang H. et al., 2018). In addition, IGF2BP1 was reported to associate with some long non-coding RNAs (lncRNAs) in cancer cells. In contrast to mRNA association, however, IGF2BP1-lncRNAs association was reported to rather serve scaffolding roles in modulating IGF2BP1 function, as for instance demonstrated for HULC and HCG11 (Hämmerle et al., 2013; Xu et al., 2017). However, although IGF2BP1-association of all to date validated target mRNAs of IGF2BP1 was confirmed by CLIP, binding information on its own proved insufficient to reveal target RNAs controlled by IGF2BP1 in respect to turnover. Inspired by recent findings on the conserved role of IGF2BP1 in promoting cancer cell cycle progression by promoting E2F-driven gene expression in cancer (Müller et al., 2020), we aim at evaluating strategies for identifying conserved effector pathways and potentially targetable effectors of IGF2BP1 in this study. This approach settles on the evaluation of altered gene expression upon IGF2BP1 depletion in six cancer cell lines derived from distinct cancer entities. The respective gene expression data were combined with publicly available cancer transcriptome data to unravel IGF2BP1-associated gene expression in primary cancers. In addition, RNA-binding information of IGF2BP1 derived by CLIP studies as well as predicted miRNA-targeting, 3’UTR properties and m^6^A-modification of candidate target mRNAs were considered. These studies confirm previous target mRNAs and IGF2BP1’s role in promoting cancer cell cycle progression. In addition, these studies suggest novel, conserved effector pathways and target transcripts of the protein, three of which (AURKA, HDLBP and YWHAZ) were validated as target mRNAs stabilized by IGF2BP1.

## 2 Materials and Methods

### 2.1 Cell Culture, Transfection, qRT-PCR and Western Blotting

A-549, ES-2, Hep-G2, MV3 and PANC-1 cells were cultured in DMEM supplemented with 10% FBS. BE (2)-C cells were cultured in EMEM:DMEM/F12 (1:1) medium supplemented with 10% FBS. All cell lines were grown at 37°C in 5% CO_2_. Cells were transfected with control siRNAs (CEL-miR-239b) or a paraloque-specific IGF2BP1-directed siRNA pool to minimize RNAi off-target effects at a final concentration of 15 nM as previously described (Müller et al., 2019) For BE (2)-C cells 25 nM siRNas were used. All other cell lines were transfected using 15 nM siRNAs as previously described (Müller et al., 2020). RNA-sequencing, qRT-PCR or Western blotting was performed 96 h (PANC-1) or 72 h (all other cell lines) post transfection referring to approximately three doublings. For RNA decay analyses, cells were treated with Actinomycin D (5 μM, Sigma Aldrich) for indicated time points 72 h upon transfection.mRNA levels were quantified by QRT-PCR based on the SYBRGreenI ® technology as previously described (Bley et al., 2020). Western blotting using the LI-COR Odyssey infrared scanning system for detection was performed as previously described in detail (Bley et al., 2020). Gene specific qRT-PCR primers are summarized in Supplementary Table S9A. Primary and secondary antibodies used for Western blotting are shown in Supplementary Table S9B.

### 2.2 RNA-Seq Library Preparation and Sequencing

#### 2.2.1 Total RNA-Seq

RNA extraction was performed using TRIzol according to manufacturer’s protocol. 1 μg of total RNA served as input for rRNA depletion using RiboCop v1.2 (Lexogen). NEBNext Ultra Directional RNA Library kit (NEB) was used for library generation. Paired-end sequencing (76 bp) of three biological replicates per condition was performed on an Illumina NextSeq 500 platform at the Deep Sequencing Facility TU Dresden.

#### 2.2.2 Poly-A RNA-Seq

72 h upon transfection cells were harvested for RNA extraction. RNA extraction was performed using TRIzol according to manufacturer’s protocol. Poly-A RNA was enriched using oligo (dT) beads. RNAs are fragmented randomly by adding fragmentation buffer. cDNA is synthesized by using random hexamers primer followed by second strand synthesis. Strand-specific double-stranded cDNA libraries were completed by size selection (250–300 bp) and PCR enrichment. Paired-end sequencing (150 bp) of three biological replicates per condition was performed on an Illumina NovaSeq 6,000 platform at Novogene (Hong Kong).

#### 2.2.3 SmallRNA-Seq

A-549, ES-2, MV3, PANC-1 and Hep-G2 cells were harvested for RNA extraction 48 h upon seeding. BE(2)-C cells were harvested 24 h upon seeding for RNA extraction. RNA extraction was performed using TRIzol according to manufacturer’s protocol. For A-549, ES-2, MV3 and PANC-1 cells, 50 ng of total RNA served as input using the NEXTflex Small RNA Library Prep Kit v3 (Bioo Scientific). Small RNA-seq libraries for HepG2 and BE(2)-C cells were prepared by Novogene (Hong Kong). Single-end sequencing [A-549, ES-2, MV3, PANC-1: 76 bp BE(2)-C, Hep-G2: 50 bp] was performed on Illumina HiSeq 2000 (A-549, ES-2, MV3, PANC-1) at the Deep Sequencing Facility TU Dresden or on an Illumina HiSeq 1500 [BE(2)-C, Hep-G2] platform at Novogene (Hong Kong). One (PANC-1) or two [A-549, BE(2)-C, ES-2, Hep-G2, MV3] biological replicates per cell line were sequenced.

### 2.3 Differential Expression Analysis

For RNA-seq data analyses low quality read ends as well as remaining parts of sequencing adapters were clipped off using Cutadapt (v 1.14). Subsequently, the processed sequencing reads were aligned to the human genome (UCSC hg38) using HiSat2 [v 2.1.0 (Kim et al., 2015)]. FeatureCounts [v 1.53 (Liao et al., 2014)] was used for summarizing gene-mapped reads. Ensembl [GRCh38.89 (Aken et al., 2017)] was used as annotation basis. Differential gene expression was determined using the R package edgeR (v 3.30.3) utilizing trimmed mean of M-values [TMM (Robinson and Oshlack, 2010; Robinson et al., 2010)] normalization. A false discovery rate (FDR) value below 0.05 was considered as threshold for the determination of differential gene expression.

### 2.4 Enrichment Analyses

Gene set enrichment analyses (GSEA) was performed using the R-package clusterProfiler v 3.16.1 (Yu et al., 2012) and MSigDB v7.1 gene sets (Liberzon et al., 2011) utilizing the fgsea algorithm and setting the exponent parameter to 0 for unweighted analyses of log2 fold change sorted gene lists from RNA-Seq data. Overrepresentation analyses (ORA) were performed using Cytoscape [v3.7.0 (Shannon et al., 2003)]and the ClueGO plugin [v2.5.7 (Bindea et al., 2009)]. Overrepresentation was determined applying the right-sided hypergeometric test, a cutoff-value for Benjamini –Hochberg corrected *p*-values of 0.05, and a minimum GO-level of four for Gene Ontology categories. We used the Gene Ontology, KEGG pathways and REACTOME pathways releases from the 8th May 2020. Enriched transcription factor sites were determined via the R-package RcisTarget [v1.8.0 (Aibar et al., 2017)]. Applied search space was 500 bp upstream to 100°bp downstream of transcription start sites of the human genome (hg38, RefSeq-r80). Only transcription factors determined with high confidence were selected.

### 2.5 TCGA Tumor Data Analyses

Normalized primary tumor expression (FPKM) and associated clinical data from the TCGA project (Cancer Genome Atlas Research Network, 2017) were obtained from the GDC data portal [Grossman et al. (2016); portal.gdc.cancer.gov]. The log-rank test and calculation of hazard ratios was implemented in an R-script according to the description in (Bewick et al., 2004). High and low expression groups were separated by the respective gene’s median RNA expression value.

### 2.6 CLIP Data Analysis

IGF2BP1 eCLIP [enhanced crosslinking and immunoprecipitation) peak data of Hep-G2 and K-562 cells were obtained from the ENCODE portal (www.encodeproject.org; (ENCODE Project Consortium, 2012); identifiers ENCFF486BXN, ENCFF976DBP and ENCFF435MEM, ENCFF701YCW, respectively]. IGF2BP1 eCLIP data of H9 cells were obtained from the Gene Expression Omnibus (GEO; sample IDs GSM2071742 and GSM2071745). Insignificant peaks (less than eight-fold enriched over input, enrichment *p*-value ≥10^−5^) were removed. Overlap between CLIP-peaks and candidate genes was determined using the intersect program of the bedtools suite [v2.25.0 (Quinlan, 2014)]. Ensembl hg19 (Cunningham et al., 2019) annotations were used as transcript references. Preprocessed AGO2 PAR-CLIP data were obtained from CLIPdb [http://lulab.life.tsinghua.edu.cn/clipdb, (Yang et al., 2015), identifiers GSM714644 and GSM714645] Overlap between CLIP-peaks and candidate genes was determined as described for eCLIP data.

### 2.7 MiRNA-mRNA Binding Prediction

Predicted miRNA-mRNA bindings were obtained by utilizing the R-package multiMiR [v1.10.0, database version 2.3.0 (Ru et al., 2014)]. All eight databases containing predicted binding information were queried (prediction cutoff 20%). If a certain miRNA-mRNA pair was obtained by at least two of these databases, it was considered as a putative interacting pair.

### 2.8 ARE Detection

For the determination of the fraction of genes with ARE-sites, ARED Plus database [https://brp.kfshrc.edu.sa/ared (Bakheet et al., 2018)] was queried using the ENSEMBL gene identifiers of the respective genes.

### 2.9 Pan-Cancer Loss-Of-Function Analysis

Pan-cancer loss-of-function CRISPR screens of 789 cancer cell lines were used for dependency analysis, using the Broad Institute Cancer Dependency Map (DepMap) portal [version 20Q3 (Meyers et al., 2017)]. Median dependency scores were calculated across available cell lines for each respective gene.

### 2.10 RNA Modification Analysis

N6-Methyladenosine (m^6^A) modification sites were identified using the RNA Modification database, RMBase [v2.0; Xuan et al. (2018)]. This database integrates public high-throughput modification sequencing data sets retrieved from the Gene Expression Omnibus (GEO), covering 13 species. Positions of m^6^A modification sites were predicted from m^6^A-seq or MeRIP-seq peaks via the RMBase workflow, resulting in 477452 human m^6^A -sites. Available sites were matched to transcripts of known human RefSeq genes (UCSC hg19). Number of identified sites were reported for each respective gene, corresponding to the transcript with maximum number of m^6^A-sites.

### 2.11 Generation of Meta-Gene Profiles

Meta-gene profiles, i.e., the distribution of relative frequencies of genomic peak data over the coding regions of target genes, were generated using the program metaProfile [v0.1 (Zhang and Yang, 2019)]. Ensembl hg19 coordinates were used as gene references. Bin size for each region (5’UTR, CDS, 3’UTR) was set to 30.

### 2.12 Drug Interaction Analysis

Drug-gene interaction analysis of target genes was performed with R package rDGIdb [v1.16.0 (Thurnherr et al., 2016)] a wrapper for the Drug Gene Interaction database (DGIdb, v3.0 (Cotto et al., 2018)[, giving access to 22 different resources. Interaction of respective genes were summarized with details about interaction types, sources of reported interactions and available literature.

### 2.13 Statistical Analysis

All statistical tests, as well as all other, not elsewhere stated statistical calculations, were performed using R (R Core Team, 2020).

## 3 Results

### 3.1 Differential Gene Expression Upon IGF2BP1 Knockdown

Aiming to identify RNAs with conserved IGF2BP1-dependent regulation in cancer cells, we considered IGF2BP1 knockdown experiments in six distinct cancer cell lines (Table 1). For IGF2BP1 depletion previously reported IGF2BP1-specific siRNA pools were used (Müller et al., 2018). Each cell line was analyzed in triplicates, including three knockdown controls and IGF2BP1 depletion studies. Differential gene expression was assessed by RNA-seq, using either ribosomal RNA depletion (total RNA) or poly-A-RNA enrichment for library preparation (Table 1). Differentially expressed genes (DEGs) were determined for each experiment (see Materials and Methods). Genes associated with false discovery rate (FDR) adjusted *p*-values below 0.05 were considered as DEGs.

**TABLE 1:** Summary of the investigated IGF2BP1 knockdown RNA-seq experiments.

Cell line	Origin	library preparation
A-549	lung adenocarcinoma	total RNA
BE (2)-C	neuroblastoma	poly-A-RNA
ES-2	ovarian clear cell adenocarcinoma	total RNA
Hep-G2	hepatoblastoma	poly-A-RNA
MV3	amelanotic melanoma	total RNA
PANC-1	pancreatic ductal adenocarcinoma	total RNA

Each experiment was conducted in a distinct cell line, libraries were either constructed by rRNA depletion (total RNA) or poly-A-RNA enrichment (poly-A-RNA).

**FIGURE 1:**
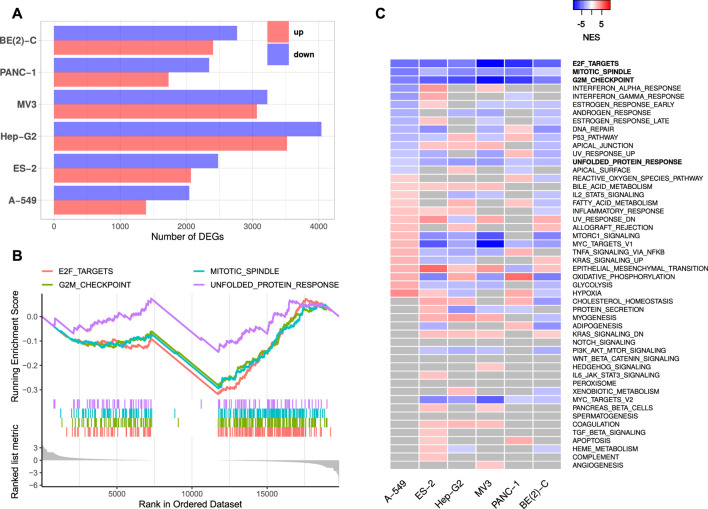
IGF2BP1 knockdown RNA-seq experiments. **(A)** Numbers of significantly up- and downregulated protein-coding genes upon IGF2BP1 knockdown in six distinct cell lines. **(B)** Exemplary GSEA running sum plot of the consistently negatively enriched hallmark gene sets from A-549 cells. **(C)** Normalized enrichment scores (NES) obtained by GSEA using hallmark gene sets. Gray values represent non-significant results (FDR ≥0.05).

We restricted our analyses to mRNAs and lincRNAs, since these classes of transcripts are generally polyadenylated (Ulitsky and Bartel, 2013) and present the only to date reported target RNA classes of IGF2BPs (Bell et al., 2013). The numbers of significantly deregulated mRNA genes ranged from 3,432 (A-549) to 7,290 (Hep-G2). In all six IGF2BP1 depletion experiments, more protein-coding genes were downregulated than upregulated (Figure 1A; Supplementary Table S1). The numbers of deregulated lincRNAs were considerably smaller, ranging from 72 (A-549) to 399 (Hep-G2). Interestingly and in contrast to mRNAs, the numbers of upregulated lincRNAs were consistently higher than the numbers of downregulated lincRNAs upon IGF2BP1 knockdown in all considered experimental studies (Supplementary Figure S1; Supplementary Table S1). This supports the notion that IGF2BP1 mainly controls mRNA abundance.

Gene set enrichment analyses (GSEA) based on RNA expression fold changes revealed pathways commonly deregulated in the six RNA-seq experiments. Out of the 50 hallmark gene sets included in the Molecular signatures database [MSigDB; Liberzon et al. (2011)], four gene sets consistently showed significant negative enrichment (normalized enrichment score (NES) <0, FDR <0.05). These gene sets contained E2F transcription factor targets, cell cycle G2M checkpoint genes, factors related to mitotic spindle assembly as well as genes upregulated during unfolded protein response (Figures 1B,C, Supplementary Table S2). Notably, no hallmark gene set was significantly positively enriched (NES >0, FDR <0.05) in all six experiments. GSEA using 184 KEGG [Kyoto Encyclopedia of Genes and Genomes, Kanehisa et al. (2017)] pathway gene sets also revealed no consistently and significantly positively enriched gene sets, but two significantly negatively enriched sets, namely KEGG_CELL_CYCLE and KEGG_SPLICEOSOME. Notably, most KEGG cancer related gene sets were scored with negative enrichment scores in all experiments, however, FDR values were not consistently below 0.05 (Supplementary Table S2). These results show, that IGF2BP1 depletion leads to a downregulation of cell cycle related genes, especially those regulated by the E2F transcription factors. This confirms the recently reported and conserved role of IGF2BP1 in the post-transcriptional enhancement of the E2F target pathway and G1 cell cycle phase shortening (Müller et al., 2020). A novel finding was the observation that the hallmark “unfolded protein response”, containing genes involved in maintaining the biosynthetic homeostasis of the endoplasmic reticulum (Schröder and Kaufman, 2005), is apparently regulated by IGF2BP1 in a conserved manner in cancer cells.

### 3.2 Genes Consistently Downregulated Upon IGF2BP1 Depletion Show Oncogenic Properties

**FIGURE 2:**
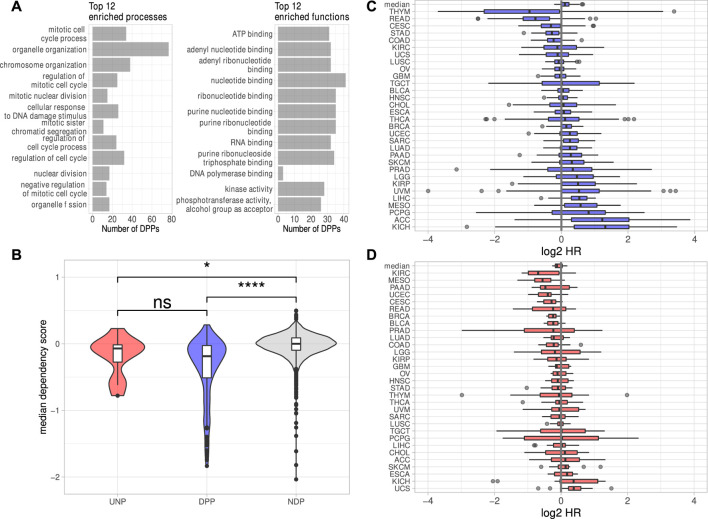
Properties of the putative IGF2BP1 targets. **(A)** Top 12 significantly (FDR <0.05) enriched biological processes and molecular functions found among the DPPs. **(B)** Dependency scores generated from data of 789 cell lines for the UNPs, DPPs and NDPs. **(C, D)** Log2 Hazard ratios (HR–high expression/low expression; patients separated by median expression values) of DPPs **(C)** and UNPs **(D)** in 31 solid tumor cohorts. The top box represents the median HRs of the respective genes in all cohorts. Statistical significance was assessed using the Mann-Whitney test. *: *p* < 0.05; ****: *p* < 0.0001; n. s.: *p* ≥ 0.05.

The investigation of DEGs observed in all six analyses revealed 238 protein-coding genes consistently down- and 42 genes consistently upregulated. Notably, the downregulation of only one lincRNA, LINC00205, was conserved over all cell lines. The lack of substantial conservation of lincRNA regulation by IGF2BP1 may be due, at least in part, to the largely tissue- or cell type-specific expression of these non-coding RNAs (Bhan et al., 2017; Ransohoff et al., 2018). Aiming to focus on conserved, IGF2BP1-dependent pan-cancer expression patterns, we calculated Spearman correlation coefficients (ρ) between the RNA expression of IGF2BP1 and the consistently deregulated protein-coding genes in RNA-seq data of 31 solid tumor cohorts obtained from the TCGA project. We expected IGF2BP1-regulated genes to be negatively correlated with IGF2BP1, when their expression increases upon IGF2BP1 knockdown and to be positively correlated when they were downregulated upon IGF2BP1 knockdown. Of the 42 protein-coding genes upregulated in all six experiments 12 (29%) showed negative values of ρ in at least 20 of the 31 analyzed tumor cohorts. We will refer to those genes as UNPs (upregulated, negatively correlated protein-coding genes) in the remainder of this article. Notably, 179 of 238 (75%) downregulated genes showed positive correlation with IGF2BP1 in at least 20 tumor cohorts. These genes will be referred to as DPPs (downregulated, positively correlated protein-coding genes). To compare properties of the consistently deregulated genes, we assembled a set of genes consistently unchanged upon IGF2BP1 knockdown. An FDR threshold of 0.95 was chosen to define no differential expression with high confidence. We obtained 3,952 protein-coding genes fulfilling this criterion in all six knockdown experiments. Since we did not filter on the magnitudes of the correlation coefficients for the determination of UNPs and DDPs, we chose all of these none DEGs, regardless of their ρ-values in relation to IGF2BP1 and refer to them as NDPs (not differentially expressed protein-coding genes). The UNPs, DPPs and NDPs are listed in Supplementary Table S3. Consistent with GSEA and the recently described role of IGF2BP1 in promoting E2F-driven gene expression (Müller et al., 2020), E2F transcription factor target genes tended to be attenuated upon IGF2BP1 knockdown. Several known E2F targets were among the DPPs, e.g., AURKA, CDK1, MKI67 or PLK1 as well as the E2F family members E2F1 and E2F2. Significant destabilization of several E2F target transcripts upon IGF2BP1 knockdown, as well as a downregulation of these transcripts upon E2F1-3 co-depletion was recently shown in PANC-1 cells (Müller et al., 2020). E2F-driven regulation of IGF2BP1 target genes was further consolidated by searching for over-represented transcription factor (TF) motifs using the RcisTarget R-package (Aibar et al., 2017). For the DPPs this analysis resulted in significantly enriched motifs of 49 distinct TFs, including E2F1-7. Enriched motifs of 152 distinct TF were found for the UNPs, including E2F1, three and four. In total, for 168 of the 179 DPPs (94%) and seven of 12 (58%) UNPs, motifs of at least one E2F transcription factor was found. Analysis of the 3,952 NDPs yieled no significantly enriched TF motif. However, since the number of NDPs was considerably larger than those of the DPPs, we randomly selected 179 NDPs and tested those genes for TF enrichments. We repeated this procedure 1,000 times. The highest number of E2F TFs found to be enriched among NDP subsamples was three. This number was achieved in two of 1,000 tests. In most cases (696/1,000) no E2F TFs were found to be enriched. Together, these findings provide further evidence for the post-transcriptional enhancement of E2F-driven gene expression by IGF2BP1. Overrepresentation analyses (ORA) using different databases (Gene Ontology, KEGG Pathways, REACTOME Pathways) supported GSEA results by revealing strong enrichment of predominantly cell cycle-related genes among the DPPs. Regarding molecular functions, a salient enrichment of RNA-binding proteins (32/179, 18%) as well as kinases (28/179, 16%) was found among the DPPs (Figure 2A, Supplementary Table S4).

To investigate essentiality of deregulated genes in respect to proliferation and survival of cancer derived cell lines, pan-cancer loss-of-function CRISPR screens were analyzed. The inspection of dependency scores using data from CRISPR knockout screens in 789 different cell lines generated by the DepMap project revealed a slight tendency of the DPPs to be ”more essential” (lower median dependency score) for proliferation than the UNPs (Figure 2B). The median dependency score of the NDPs, however, was even a bit higher but remained essentially at zero in median, suggesting that the vast majority of proteins encoded by these transcripts barely serve roles in promoting or impairing tumor cell survival, at least *in vitro*. Despite only mild differences, these analyses support the notion that IGF2BP1 tends to stabilize transcripts encoding factors promoting tumor cell vitality *in vitro*. This was further evaluated by the inspection of prognostic relevancies of the UNPs and DPPs. Hazard ratios (HR) between high and low RNA expression were determined for the respective genes in 31 TCGA tumor cohorts. This revealed that high RNA expression of the DPPs tended to be associated with dismal prognosis, as indicated by median log2 HR values greater then 0 (Figure 2C). The opposite was observed for UNPs, since these rather tended to be associated with improved outcome at elevated expression (Figure 2D). Despite the general trend of most DPPs being associated with reduced survival probability, when expressed at higher levels, in some tumor cohorts, especially thymoma (THYM) and rectum adenocarcinoma (READ), high expression of most of the DPPs was associated with better survival (Figures 2C,D, Supplementary Table S5). Thus, in conclusion mRNAs consistently downregulated upon IGF2BP1 depletion and, in addition, positively correlated with IGF2BP1 RNA expression in a majority of solid tumors, i.e., the DPPs, tend to promote tumor cell vitality *in vitro* and appear associated with adverse patient outcome when highly expressed. The opposite is observed for the comparatively small number of transcripts consistently upregulated upon IGF2BP1 depletion and generally negatively correlated with IGF2BP1 expression (UNPs) in primary cancers. This supports the notion, that IGF2BP1 drives tumor progression primarily by stabilizing mRNAs encoding pro-oncogenic proteins, mostly factors serving roles in tumor cell proliferation and cell cycle progression.

### 3.3 IGF2BP1 Controls Conserved Candidate Target mRNAs in a Mostly 3’UTR-Dependent Manner

**FIGURE 3:**
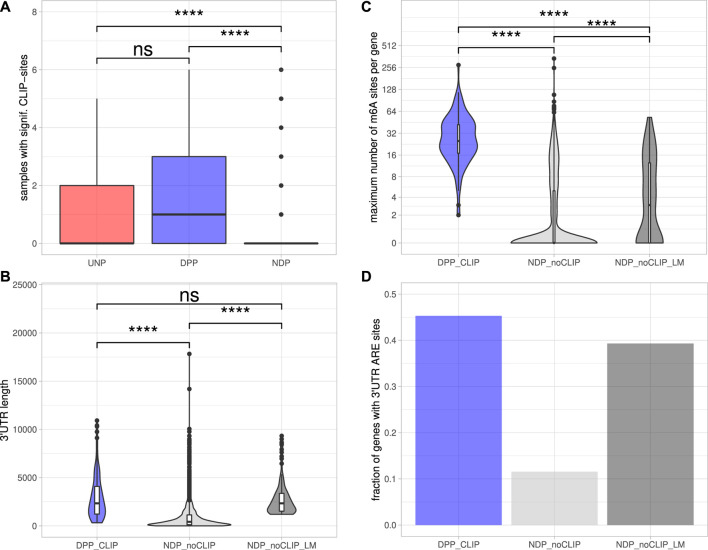
Post-transcriptional regulation of putative IGF2BP1 targets.**(A)** Distribution of the number of samples with significant eCLIP-sites for the indicated gene sets. **(B)** Distribution of maximum 3’UTR lengths. **(C)** Number of m^6^A-sites per gene. The numbers refer to the transcript forms with the most reported methylation sites of the respective genes. **(D)** Fraction of genes with predicted AU-rich elements (AREs) in their 3’UTRs. Statistical significance was assessed using the Mann-Whitney test. ****: *p* < 0.0001; n. s.: *p* ≥ 0.05.

To determine DPPs and UNPs that might be regulated via direct binding of IGF2BP1, eCLIP [enhanced crosslinking and immunoprecipitation; Van Nostrand et al. (2016)] binding studies performed in Hep-G2, chronic myeloid leukemia-derived K-562 and the human embryonic stem cell line H9 (Conway et al., 2016; Van Nostrand et al., 2020) were evaluated. Significant eCLIP-sites (eight-fold enriched over input, enrichment *p*-value <10^−5^) were found for 117 of 179 DPP genes (65%) and five of the 12 UNPs (42%) in at least one of the CLIP-samples. In contrast, for only 191 of the 3952 NDP genes (5%) significant eCLIP-sites were identified. Considering the number of distinct CLIP samples with significant peaks for a certain gene further revealed that DPPs on average were found in more samples than UDPs and NDPs (Figure 3A). Notably, the 117 DPPs associated with significant CLIP-sites still contained representatives of all four gene sets found to be consistently enriched upon IGF2BP1 knockdown (Figure 1C). For example, the kinases AURKA, CDK1 and PLK1 as well as the marker of proliferation Ki-67 (MKI67) were found in the sets representing E2F target and G2M checkpoint genes. AURKA, CDK1 and PLK1 were also found in the gene set representing genes important for mitotic spindle assembly. With E2F1, SRF, MAPK1, SIRT1 and MKI67 our studies identified five previously validated target mRNAs stabilized by IGF2BP1 (Gutschner et al., 2014; Müller et al., 2019; Müller et al., 2020). Accordingly, the 117 DPPs associated with significant eCLIP-sites, denoted as DPP_CLIP_, were considered as prime candidates of conservedly stabilized target mRNAs of IGF2BP1 in cancer cells. Properties regarding post-transcriptional regulation of these mRNAs by IGF2BP1 outlined in the following were compared to the 3,761 NDPs that showed no significant eCLIP-sites in any of the analyzed CLIP samples (NDP_noCLIP_). In addition, we selected 117 of these NDP_noCLIP_ genes with a similar maximum 3’UTR length distribution than observed for the 117 DPP_CLIP_ genes, in order to examine if differences between NDP_noCLIP_ and DPP_CLIP_ transcripts, regarding regulatory elements, were merely attributed to longer 3’UTRs of the DPP_CLIP_ transcripts. The set of NDP_noCLIP_ genes with a length-matched 3’UTR length distribution is denoted as NDP_noCLIP_LM_.

#### 3.3.1 3’UTR Length Properties of Stabilized IGF2BP1 Candidate Target mRNAs

The 5’ and 3’ untranslated regions (5’ or 3’UTRs) of mRNAs are essential for the regulation of mRNA fate in respect to their subcellular sorting, translation and turnover control facilitated by trans-acting factors including RBPs and non-coding RNAs, primarily miRNAs. The length of 3’UTRs has substantially expanded during the evolution of higher organisms and correlates with cellular complexity of organisms (Mayr, 2019). This suggests that transcripts having long 3’UTR sequences are more likely to bear regulatory cis-elements and thus are subjected to complex regulations by trans-acting factors. To evaluate 3’UTR properties of IGF2BP1 candidate target mRNAs, we considered the respective transcript with the longest 3’UTR reported in the ENSEMBL [v89; Cunningham et al. (2019)] database of each candidate. Inspection of the 3′UTR lengths revealed that the DPP_CLIP_ transcripts tend to have significantly longer 3’UTRs (2,338 nt median length) than the NDP_noCLIP_ mRNAs (419 nt median length; Figure 3B), supporting the hypothesis that IGF2BP1 stabilizes its targets in a mostly 3’UTR-dependent manner, as previously shown for the majority of to date reported target mRNAs stabilized by IGF2BP1 (Mongroo et al., 2011; Zirkel et al., 2013; Busch et al., 2016; Müller et al., 2018; Müller et al., 2019). Median 3’UTR length of the NDP_noCLIP_LM_ transcripts was chosen to be close to the median of the DPP_CLIP_ transcripts (2,337 nt).

#### 3.3.2 N6-Methyladenosine (m^6^A) Modification of Stabilized IGF2BP1 Candidate Target mRNAs

Among the plethora of known RNA modifications, N6-methyladenosine (m^6^A) is the most prevalent internal modification of mRNA in eukaryotic cells (He et al., 2019). This modification is sharply enriched in 3’UTRs, in proximity of the stop codon and within internal long exons. M^6^A methylation affects almost every aspect of RNA metabolism, including RNA expression, splicing, nuclear export, translation, decay and RNA-protein interactions. Deregulation of either m^6^A-modification or m^6^A-reader expression was shown to play an important role in tumor initiation and progression (Sun et al., 2019). IGF2BP1 was recently identified to be an m^6^A-reader, associating preferentially with N6-methyladenosine modified target mRNAs and the m^6^A-enhanced mRNA association of IGF2BP1 was shown to result in elevated mRNA stabilization and enforced expression of MYC and SRF transcripts (Huang H. et al., 2018; Müller et al., 2019). The distribution of m^6^A-sites determined in public high-throughput m^6^A-modification data sets (m^6^A- or MeRIP-seq) demonstrated that all 117 DPP_CLIP_ mRNAs contain experimentally determined m^6^A-sites. In contrast, in only less than 40% of the 3,761 NDP_noCLIP_ transcripts, this modification was reported. Furthermore, the numbers of distinct sites found on the transcripts differed considerably. The median number of m^6^A-sites per transcript was 25 for the DPP_CLIP_ and 0 for the NDP_noCLIP_ mRNAs. Among the 3’UTR length-matched NDP_noCLIP_LM_ transcripts, 64 (55%) had reported m^6^A-sites and the median number of those sites per transcript was three and thus about 8-fold lower than observed in DPP_CLIP_ transcripts (Figure 3C).

#### 3.3.3 AU-Rich Element (ARE) Content of Stabilized IGF2BP1 Candidate Target mRNAs

The stability of mRNAs is frequently associated with the occurrence of AU-rich elements (AREs) in the respective 3’UTRs. AREs are observed in approximately 8% of human mRNAs (Khabar, 2005). These mRNAs encode proteins involved in the cellular stress responses, immune cell cross-talk and activation, apoptosis, cancer progression and most notably cell cycle regulation (Bakheet et al., 2006). In general, AREs are considered to serve essential roles for the association of stabilizing as well as destabilizing transacting factors, e.g., ELAVL1 (HuR), influence miRNA-dependent regulation of the respective transcripts (Mayr, 2019) and mostly indicate mRNAs with comparably short half-life. ARE occurrence and frequency in candidate target mRNAs of IGF2BP1 was evaluated by predicting AREs via the ARED Plus database (Bakheet et al., 2018). These analyses revealed AREs in 45% of the DPP_CLIP_ 3’UTRs, whereas only in 12% of the NDP_noCLIP_ 3’UTRs AREs could be found. In 39% of the NDP_noCLIP_LM_ transcripts AREs were found (Figure 3D). Thus, although ARE occurrences seem to be strongly correlated to 3’UTR length, IGF2BP1 targets seem to be slightly enriched for these stability determinants. This observation supports the notion that IGF2BP1 target transcripts are overall less stable, that their encoded proteins are critically involved in cancer progression and cell cycle control and that IGF2BP1 may influence ARE-dependent regulation of mRNA fate.

#### 3.3.4 Regulation of Stabilized IGF2BP1 Candidate Target mRNAs by miRNAs

**FIGURE 4:**
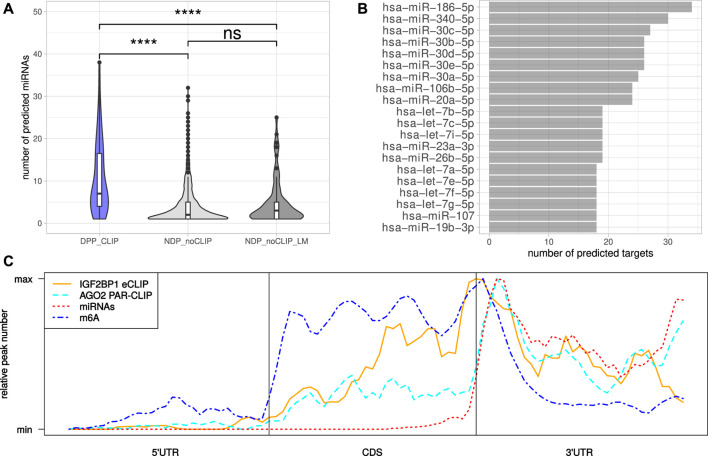
MiRNAs mediated regulation and distribution of regulatory cis-elements. **(A)** Distribution of the number of distinct miRNAs predicted to bind the DPP_CLIP_ and NDP_noCLIP_ genes. **(B)** Top 20 miRNAs predicted to bind to the most DPP_CLIP_ genes. **(C)** Meta-gene profiles of IGF2BP1-eCLIP, AGO2-PAR-CLIP, predicted miRNA (TargetScan) and reported m^6^A sites of the DPP_CLIP_ genes. Statistical significance was assessed using the Mann-Whitney test. ****: *p* < 0.0001.

The miRNA-directed inhibition of mRNA translation and induction of their decay, essentially relies on the respective 3’UTRs. Accordingly, the tendency of the DPP_CLIP_ transcripts for longer 3’UTRs and the reported role of IGF2BP1 in controlling mRNA fate in a largely miRNA-dependent manner suggested more frequent regulation by miRNAs (Elcheva et al., 2009; Jønson et al., 2014; Busch et al., 2016; Müller et al., 2018; Müller et al., 2019). This was evaluated by monitoring the expression of miRNAs by small RNA-seq in the six cell lines considered in this study (Supplementary Table S6). Putative regulation of the candidate target mRNAs by miRNAs expressed in the respective cell lines was investigated in eight databases containing predicted miRNA target engagement information. The threshold of mRNA engagement was prediction of miRNA targeting by at least two of the databases. Out of 119 miRNAs with average expression <100 CPM (counts per million mapped reads, Supplementary Figure S2A) in at least four cell lines, 99 miRNAs were predicted to target at least one DPP_CLIP_. On the other hand, of the 117 DPP_CLIP_, 103 (88%) were predicted to be regulated by at least one of these 99 miRNAs (Supplementary Table S7). When considering the numbers of distinct miRNAs targeting transcripts of a certain gene, significantly more miRNAs were predicted to bind DPP_CLIP_ than NDP_noCLIP_ as well as NDP_noCLIP_LM_ transcripts (Figure 4A). Thus, candidate IGF2BP1 targets appeared to be more susceptible to miRNA-mediated regulation. Among the miRNAs predicted to bind the most DPP_CLIP_ mRNAs were miR-186, miR-340 as well as several members of the miR-30 miRNA-family (Figure 4B). These miRNAs were also predicted to bind the most NDP_noCLIP_ transcripts, however, the fractions of transcripts predicted to be targeted by miRNAs were much smaller (Supplementary Figure S2B). For example, 34 (29%) of DPP_CLIP_ mRNAs were predicted targets of miR-186–5p, whereas this was only observed for 161 (4%) of the NDP_noCLIP_ and 18 (15%) of the NDP_noCLIP_LM_ transcripts. Notably, 32 DPP_CLIP_ mRNAs (27%) were predicted targets of the let-7 miRNA-family. This finding is in agreement with previous reports of let-7 dependent regulation of HMGA2 and LIN28B expression and an aggressive tumor cell phenotype by IGF2BP1 (Busch et al., 2016). Indeed, several of the predicted interactions have already been validated experimentally, supporting the cogency of our *in-silico* analysis. e. g., targeting of the IGF2BP1 mRNA by several members of the let-7 family has been reported in A-549 and ES-2 cells (Boyerinas et al., 2008; Busch et al., 2016). Furthermore, an association between LIMK1 and miR-27b was shown for colorectal cancer and A-549 cells (Wan et al., 2014; Chen et al., 2017). Additional predicted interactions that already have been validated include the association between KIF11 and miR-30a (Wang et al., 2020), between E2F1 and miR-93 (Bao et al., 2020) and between AURKA and miR-186 (Schmittgen, 2019).

#### 3.3.5 Spatial Distribution of Regulatory Cis-elements in IGF2BP1 Candidate Target mRNAs

Inspection of the IGF2BP1 eCLIP-sites in DPP_CLIP_ mRNAs, represented by meta-gene profiles, revealed, that IGF2BP1 binding peaks around the stop codon, but also shows substantial binding in the coding sequence as well as distal 3’UTRs (Figure 4C). The investigation of AGO2 PAR-CLIP-sites identified in HEK-293 cells (Kishore et al., 2011), showed preferential binding in the 3’UTR with AGO2 peaks downstream of the stop codon. This peak shows high congruence with coordinates of predicted miRNA binding provided by TargetScan (Agarwal et al., 2015). M^6^A-sites reported by RMBase also peak around the stop codon with its maximum between those of IGF2BP1 and AGO2. These considerations suggest, that although IGF2BP1 is considered to shield its target mRNAs from miRNA mediated decay and might bind in an m^6^A-dependent manner, IGF2BP1 binding sites do neither strictly overlap with sites of miRNA targeting nor m^6^A-modification. The inspection of reported IGF2BP1 binding motifs derived from RNA-association studies (Hafner et al., 2010; Conway et al., 2016), in the respective longest 3’UTR sequences of the DPP_CLIP_ and NDP_noCLIP_ mRNAs, revealed a substantially higher number of absolute occurrences of these motifs in 3’UTR sequences of the DPP_CLIP_ transcripts. However, the 3’UTRs of the DPP_CLIP_ transcripts were significantly longer than those of the NDP_noCLIP_ transcripts and thus, short (4mers) sequence motifs are expected to occur more frequently in these sequences by chance. Accordingly, also control motifs were found more frequently in DPP_CLIP_ 3’UTR sequences (Supplementary Figure S3A). After normalizing the absolute motif occurrences to considered sequence lengths, only slight differences in the frequencies of the proposed IGF2BP1 binding motifs were observed (Supplementary Figure S3B). Similar results were obtained by considering entire mRNA sequences (Supplementary Figure S3C). This might suggest, that the binding specificity of IGF2BP1 with its six RNA binding domains cannot be fully recapitulated by putative, four nucleotide long motifs.

### 3.4 Druggability of IGF2BP1 Candidate Effectors

IGF2BP proteins have been reported to influence the sensitivity of chemotherapeutics, as for instance demonstrated in neuroblastoma cell lines, where IGF2BP1 promotes resistance towards doxorubicin (Bell et al., 2015). Moreover, we recently demonstrated that IGF2BP1-RNA association is impaired by the small molecule drug BTYNB, interfering with the IGF2BP1-directed post-transcriptional super-enhancement of E2F-driven gene expression *in cellulo* and in mouse tumor models (Müller et al., 2020). In agreement, BTYNB impaired tumor cell vitality in strong synergy with the CDK4/6 inhibitor Palbociclib. Thus, it is tempting to speculate that the enhancement of oncogenic factor expression by impairing mRNA decay promotes chemoresistance and that inhibiting IGF2BP1-RNA association by BTYNB improves chemosensitivity. Aiming to reveal candidate therapeutics potentially acting in synergy with BTYNB, we analyzed DPP-encoding genes for known and predicted drug interactions in 22 databases. These analyses indicated that 10 of the 179 DPPs (IRAK1, PIP4K2C, CAMKK2, ICK, STK10, LIMK1, CIT, AURKA, CDK1 and PLK1) were found to be inhibited by the kinase inhibitor fostamatinib or, more specifically, its pharmacologically active metabolite R406 (Supplementary Table S8). This compound was initially described as inhibitor of the spleen tyrosine kinase [SYK; Braselmann et al. (2006)], however, like most kinase inhibitors, also R406 was reported to impair a variety of other kinases and may even engage with non-kinase targets (Rolf et al., 2015). Thus, R406 appears to be a reasonable candidate drug for further investigating, whether BTYNB and inhibitors of potential cell-cycle related IGF2BP1 targets, like AURKA, PLK1 or CDK1 exhibit synergistic effects, similar to those shown for Palbociclib. Future studies will have to reveal the potential benefit of combined inhibition of IGF2BP1-RNA association and pro-oncogenic factors enhanced by IGF2BP1 in cancer cells in a conserved manner.

### 3.5 Validation of Novel IGF2BP1 Targets

**FIGURE 5:**
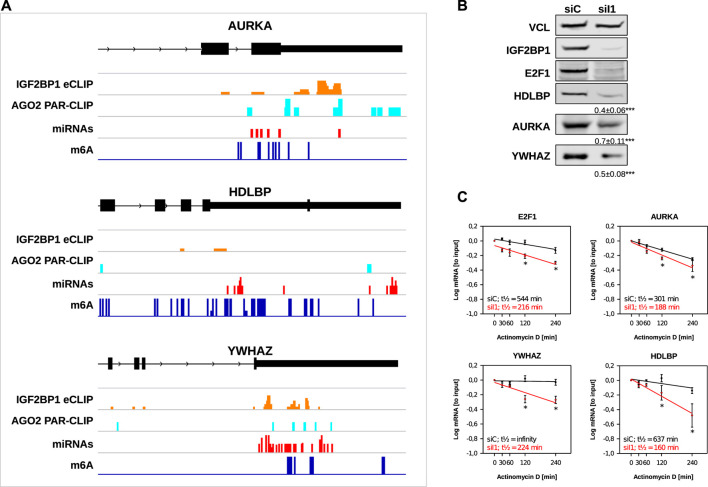
Novel IGF2BP1 candidate target transcripts. **(A)** Distribution of IGF2BP1 eCLIP, AGO2 PAR-CLIP, predicted miRNA binding and m^6^A sites along the last exons (black boxes), including 3’UTR (narrow black boxes) of three selected putative IGF2BP1 target mRNAs **(B)** Representative Western blot analyses of selected IGF2BP1 candidate target transcripts upon IGF2BP1 depletion in ES-2 cells. Vinculin (VCL) served as a loading and normalization control. Average fold change and standard deviation of protein levels, determined in three independent analyses are indicated in bottom panels. **(C)** mRNA decay of selected IGF2BP1 candidate target transcripts was monitored by RT-q-PCR in control- (siC; black) and IGF2BP1-depleted (siI1; red) ES-2 cells upon indicated time of Actinomycin D treatment. Error bars indicate standard deviation. Average mRNA half-life, determined in three independent studies is indicated. Statistical significance was assessed by Student’s *t*-test. *: *p* < 0.05.

Our *in silico* studies confirmed recently reported and revealed a variety of novel candidate target mRNAs of IGF2BP1 in cancer cells. To further evaluate our analyses, we chose three novel candidate target mRNAs, AURKA, HDLBP (alias vigilin) and YWHAZ (alias 14-3-3-ζ). The recently reported E2F1 mRNA served as positive control. All three novel candidate target mRNAs showed consistent downregulation in the investigated cancer cell models upon IGF2BP1 knockdown and their RNA expression was positively associated with IGF2BP1 expression across solid cancers. Moreover, for all three of these transcripts binding of IGF2BP1 was reported by CLIP studies and they contain sites of predicted miRNA targeting, AGO2 PAR-CLIP sites as well as m^6^A sites (Figure 5A). ARED Plus predicted AREs were only found in intronic parts the pre-mRNAs of these genes. To further evaluate IGF2BP1-dependent regulation, protein abundance was monitored in ES-2 ovarian cancer cells upon IGF2BP1 depletion. Consistent with decreased steady mRNA levels all three novel candidate effectors and E2F1 were downregulated by IGF2BP1 knockdown (Figure 5B). If the respective mRNAs are potentially stabilized by IGF2BP1 was evaluated by monitoring mRNA decay in ES-2 cells depleted for IGF2BP1 and treated with Actinomycin D (ActD). In contrast to the control mRNA GAPDH (Supplementary Figure S4), all three novel IGF2BP1 target mRNAs showed substantial reductions of their half-lifes in the presence of ActD (Figure 5C). Notably, we could not observe an increase of the mRNA half-life of any of the five UNPCLIP transcripts (Supplementary Figure S4), suggesting that IGF2BP1 binding does not lead to a stabilization of these transcripts. Further in depth investigation is required to evaluate suggested miRNA-, m^6^A- and potentially ARE-dependent regulation of the three target mRNA of IGF2BP1. However, evaluation of these candidate mRNAs provides strong evidence for the validity of the *in silico* studies and support the notion that IGF2BP1 is a conserved, post-transcriptional enhancer of pro-oncogenic factors in cancer cells due to primarily 3’UTR dependent stabilization of target mRNAs.

## 4 Discussion

In the here presented study we present an analysis pipeline for the identification of conserved cancer hallmark pathways influenced by IGF2BP1 by stabilizing target mRNAs encoding pro-oncogenic factors. Emanating from altered gene expression upon IGF2BP1 in a panel of six cancer cell lines and determining IGF2BP1-associated gene expression in publicly available cancer transcriptome data, we compile a list of prime candidate target mRNAs stabilized by IGF2BP1 in a conserved manner in solid cancer. Gene set enrichment analyses (GSEA) of candidate target mRNA of IGF2BP1 support a pivotal role of the protein in cancer cell cycle progression, but furthermore highlight cancer hallmark pathways influenced by IGF2BP1. The implementation of information on IGF2BP1-RNA association, m^6^A-modification, predicted regulation by miRNAs, AU-rich elements (AREs) content and 3’UTR length, reveal enriched features of target mRNAs stabilized by IGF2BP1. The majority these shows preferred association of IGF2BP1 in the 3’UTR close to the stop codon. In addition, most stabilized target transcripts present comparatively long 3’UTRs, m^6^A-modification in 5’-proximity to IGF2BP1 binding and targeting by miRNAs and AGO2 in the 3′-vicinity to IGF2BP1 association. Moreover, many of these target mRNAs are predicted to contain AREs in their 3’UTR and some encode proteins targetable by cancer therapeutics. To prove the validity of these in silico evaluations, we validate three novel target mRNAs by demonstrating that IGF2BP1 promotes the expression of AURKA, HDLBP, and YWHAZ in cancer cells by impairing decay of the respective mRNAs. In conclusion, our studies provide a comprehensive view on conserved roles of IGF2BP1-dependent mRNA stabilization in cancer. This information allows pursuing the evaluation of targetable IGF2BP1 effectors to test the potential benefit of inhibiting IGF2BP1-RNA association and combined treatment with effector inhibition in cancer therapy. In agreement with recent studies indicating a conserved role of IGF2BP1 in controlling cancer cell cycle progression by promoting E2F-driven gene expression (Müller et al., 2020), our studies identify cell cycle progression cancer hallmark gene sets as the most consistently deregulated pathways upon IGF2BP1 depletion. In further support of a post-transcriptional super enhancer function of IGF2BP1 in E2F-driven gene expression, E2F1/2 as well as the E2F-driven transcripts MKI67 are observed among the top candidate target mRNAs. In addition, novel, previously not investigated E2F-driven transcripts like AURKA are suggested by our investigation. In support of *in silico* studies, we confirm that AURKA mRNA as well as protein abundance is substantially reduced by IGF2BP1 depletion due to enhanced decay of the AURKA mRNA. Aurora kinases (AURKs) are key mitotic protein kinases guiding cell cycle progression by the spatiotemporal control of the onset and progression of mitotic chromosomal segregation, reviewed in Willems et al. (2018). All three aurora kinases, AURKA-C, serve oncogenic roles in cancer by promoting cell cycle progression, cancer cell survival, and promoting MYC/MYCN expression and activity (Otto et al., 2009; Dauch et al., 2016; Willems et al., 2018). Accordingly, the AURKs, in particular AURKA and B were proposed as promising targets for cancer therapy, e.g. in lung cancer treatment (Galetta and Cortes-Dericks, 2020), where AURK inhibition is currently evaluated in clinical trials. In view of recent findings, indicating therapeutic benefit of combined inhibition of IGF2BP1-RNA binding by BTYNB and the CDK4/6 inhibitor palbociclib, the identification of conserved upregulation of AURKA by IGF2BP1 suggests that IGF2BP1 inhibition may improve AURKA-inhibition in combined therapies (Müller et al., 2020). Notably, although our studies do not suggest high conservation of direct regulation of MYC mRNA turnover in cancer cells, as previously reported (Müller et al., 2020), MYC/N-driven gene expression is one of the most frequently deregulated pathways upon perturbing IGF2BP1 abundance in cancer cells. In view of the identification of AURKA regulation by IGF2BP1 highlight yet another interconnection of previously reported IGF2BP1 and MYC/N-driven gene expression in cancer (Köbel et al., 2007; Bell et al., 2015). Collectively, this emphasizes a broad, multilayered role of IGF2BP1 in promoting key cancer hallmark pathways like MYC/N- and E2F-driven gene expression. Although not comprised in any cancer hallmark gene set, we decided to evaluate regulation of HDLBP expression by IGF2BP1 and confirmed that IGF2BP1 promotes HDLBP expression by mRNA stabilization. The multi-KH domain containing RNA-binding protein HDLBP, also termed vigilin, has been reported to serve pathophysiological roles in cancer and cardiovascular diseases, reviewed in Cheng and Jansen (2017). In cancer, upregulation of HDLBP expression, e.g. in liver cancer (Yang et al., 2014), has been reported and the protein was proposed to promote proliferation by enhancing G1/S cell cycle transition (Zhou et al., 2019). The latter, more specifically the shortening of G1 cell cycle phase length by promoting G1/S transition, was identified as a key function of IGF2BP1 in promoting cancer cell cycle progression (Müller et al., 2020). This suggest that next to regulating E2F-driven gene expression, IGF2BP1 may influence this cell cycle checkpoint also by promoting the expression of HDLBP. Future studies have to reveal if and how IGF2BP1 influences other HDLBP-dependent regulation of gene expression. Of particular interest in this respect are recent reports suggesting that HDLBP influences IGF2 synthesis by modulating imprinting via association with CTCF and the non-coding H19 RNA (Yu et al., 2018), a reported target RNA of IGF2BP1 in cancer cells, reviewed in Zhang et al. (2017). A novel finding of our studies is an apparently conserved role of IGF2BP1 in modulating the unfolded protein response in cancer cells. One prime candidate target mRNA comprised in the respective hallmark gene set is the YWHAZ transcript, encoding the 14-3-3 ζ protein. Members of the 14-3-3 protein family associate with hundreds of phosphorylated proteins and have been implicated in a variety of cellular processes by serving as regulatory adaptor molecules modulating protein function, folding and decay, reviewed in Fan et al. (2019). In cancer, the 14-3-3ζ is substantially upregulated in various carcinomas, where it has been proposed to enhance cancer cell survival through binding of the p85 subunit of the PI3K resulting in activation of AKTs and/or by impairing tumor cell senescence in a STAT3/SKP2/p27-dependent manner (Neal et al., 2012; Lee et al., 2014). In addition, 14-3-3ζ was proposed to promote cytoplasmic accumulation of FOXO3a, modulates Wnt5A/ROR1 signaling and contributes to the switch from tumor suppressor activity of TGFβ to a pro-metastatic and pro-proliferative activity reviewed in Fan et al. (2019). Accordingly, YWHAZ presents yet another conserved bona fide pro-oncogenic effector of IGF2BP1. By enhancing YWHAZ expression, IGF2BP1 promotes an highly proliferative, pro-survival and metastatic tumor cell phenotype, as previously described as the main conserved role of IGF2BP1 in cancer (Müller et al., 2018). At the target mRNA level our studies provide comprehensive support for a largely 3’UTR-, m^6^A-, and miRNA-dependent regulation of target mRNA turnover. However, the investigation of metagene profiles of candidate target mRNAs stabilized by IGF2BP1 suggest that IGF2BP1 predominantly associates in between preferred sites of m^6^A-modification and miRNA targeting. This suggests that enhanced association of IGF2BP1 with m^6^A-modified target mRNAs does not necessarily rely on direct binding to modified nucleotides, but may rather involve m^6^A-dependent structural rearrangements of binding regions, favoring or stabilizing IGF2BP1-association. In respect to inhibiting miRNA-directed regulation, our studies provide further support that IGF2BP1 not necessarily directly masks miRNA-targeting sites (MTS). This is suggested by the fact that IGF2BP1 impairs a variety miRNAs with quite distinct seeds, that it associates preferentially in the 5’-proximity of MTSs and that its associates with target mRNAs in cytoplasmic mRNPs devoid of miRNAs and RISC components like AGO2 (Busch et al., 2016). These findings support the notion that IGF2BP1 is a multi-versatile inhibitor of miRNA-directed downregulation and that the control of target mRNA fate largely relies on the portfolio of expressed miRNAs. A novel observation is that IGF2BP1 target mRNAs stabilized by IGF2BP1 appear enriched for AU-rich elements. This supports the notion that IGF2BP1 preferentially, but not exclusively, stabilizes short-lived mRNAs. Moreover, this finding indicates a potential cross-regulation with other pro-oncogenic RBPs controlling mRNA turnover in an ARE-dependent manner like for instance ELAVL1, associating with IGF2BP1 in an RNA-dependent manner (Weidensdorfer et al., 2009; Wächter et al., 2013).

In conclusion, our studies reveal a potent analysis pipeline for the identification of conserved, pro-oncogenic effectors and hallmark pathways regulated by IGF2BP1 in cancer. The further evaluation of IGF2BP1’s role in modulating the expression of such effectors like AURKA will unravel novel avenues to pursue the inhibition of IGF2BP1-mRNA association in combinatorial treatment strategies to improve cancer patient outcome.

## Data Availability

The datasets presented in this study can be found in online repositories. The names of the repository/repositories and accession number(s) can be found below: https://www.ncbi.nlm.nih.gov/geo/, GSE161101.
