# Supplementary material for: IGF2BP1, a Conserved Regulator of RNA Turnover in Cancer
Source: Front Mol Biosci. 2021 Mar 22;8:632219. doi: 10.3389/fmolb.2021.632219 (PMC8019740; doi:10.3389/fmolb.2021.632219)
Supplement: Supplementary file 2 [file datasheet2.pdf]

Supplementary Material

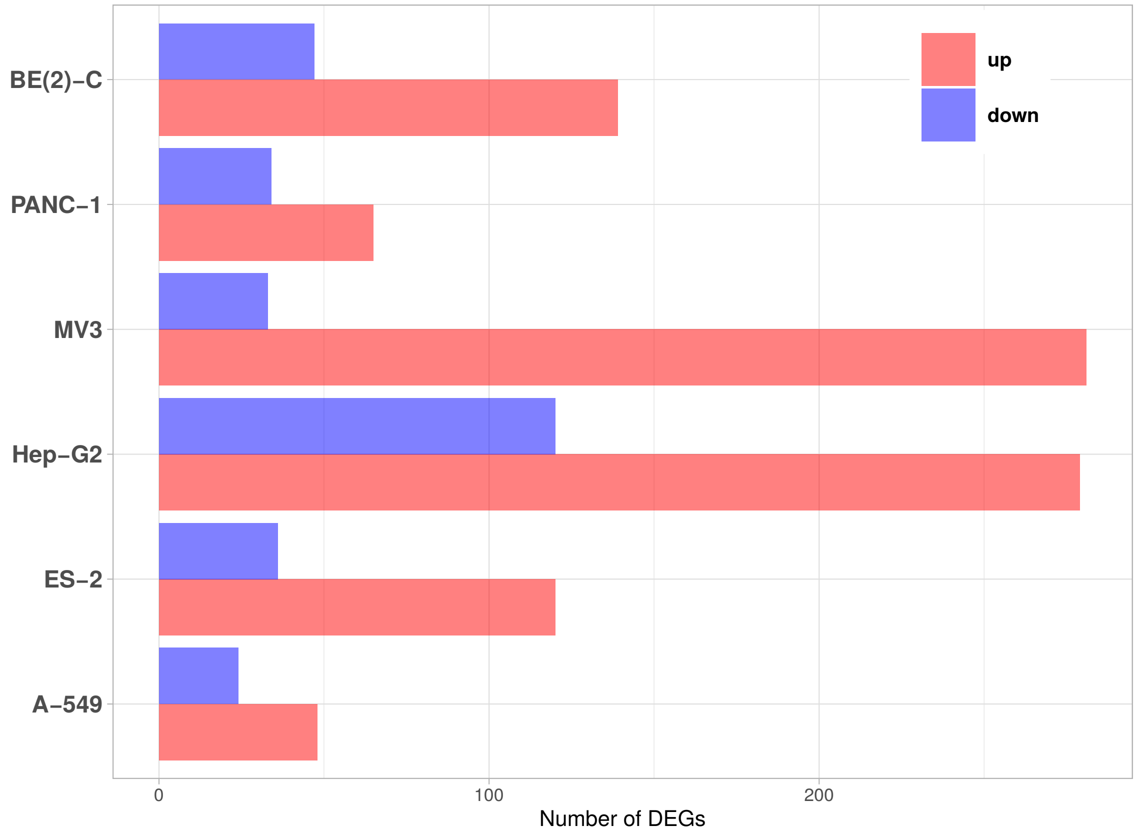

Figure S1. Numbers of significantly up- and downregulated lincRNA genes upon IGF2BP1 knockdown in six distinct cell lines.

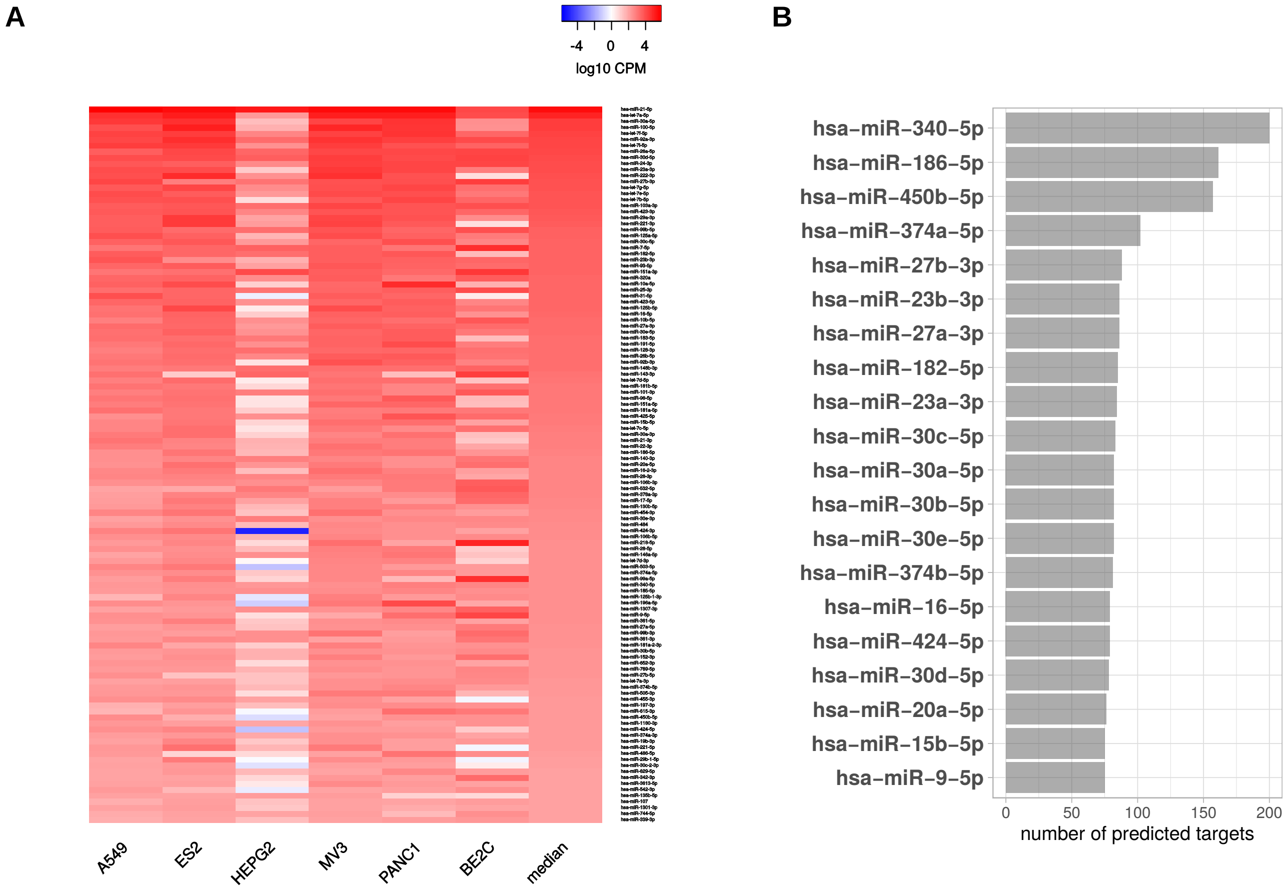

Figure S2. MiRNA expression and target predictions. **(A)** Normalized expression ($\log_{10}$ CPM) of miRNAs with a minimum average expression of 100 CPM in at least four of the six indicated cell lines. **(B)** Top 20 miRNAs predicted to bind to the most NDP_{noCLIP} genes.

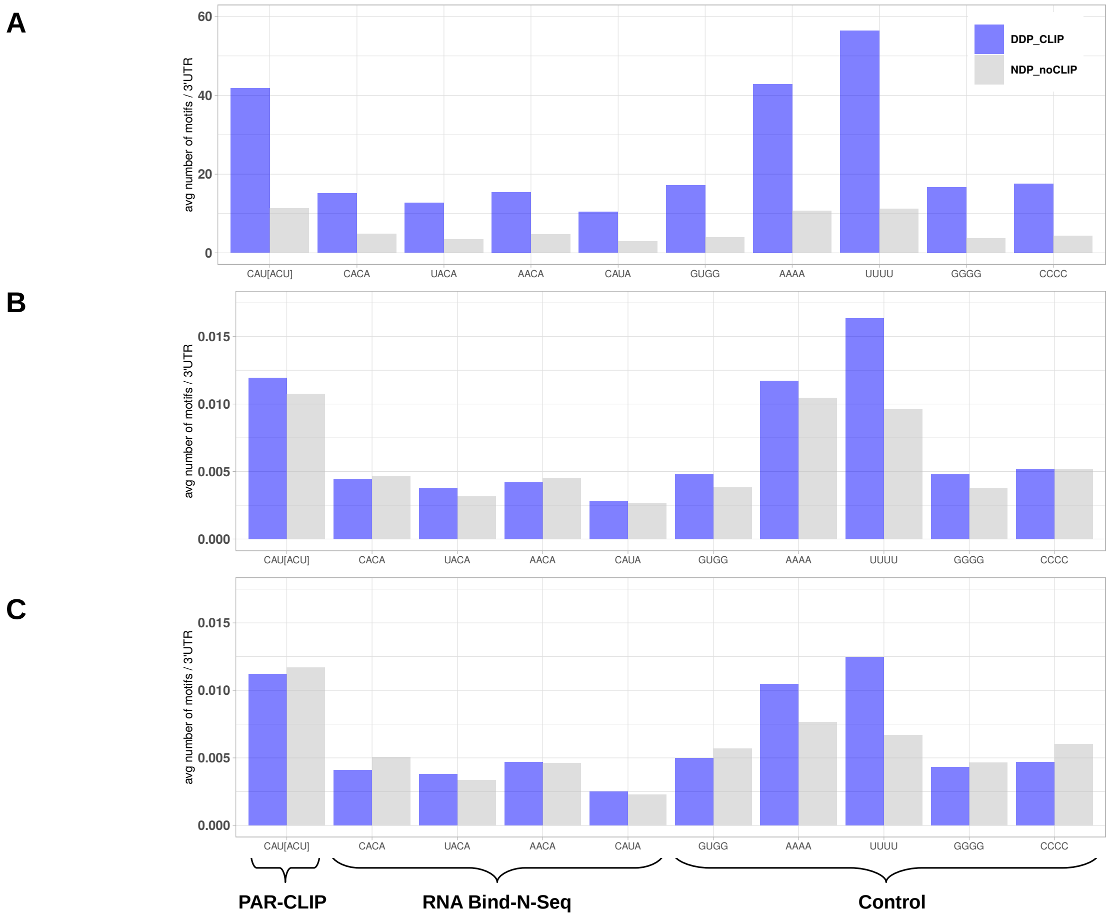

Figure S3. Frequencies of IGF2BP1 binding motifs in target gene sequences. Occurrences of the CAUH (H = A, U, or C) motif derived from PAR-CLIP-data (Hafner et al., 2010) and 3 motifs derived from RNA Bind-N-Seq data (Conway et al., 2016) as well as 4 nt long motifs unrelated to the propoese IGF2BP1 binding motifs (Control). **(A)** Average numbers of proposed IGF2BP1 RNA binding motifs in the longest DPP_{CLIP} and NDP_{noCLIP} 3'UTR sequences. **(B)** Average numbers of proposed IGF2BP1 RNA binding motifs in the longest DPP_{CLIP} and NDP_{noCLIP} 3'UTR sequences normalized to sequence lengths. **(C)** Average numbers of proposed IGF2BP1 RNA binding motifs in the longest DPP_{CLIP} and NDP_{noCLIP} mRNA sequences normalized to sequence lengths

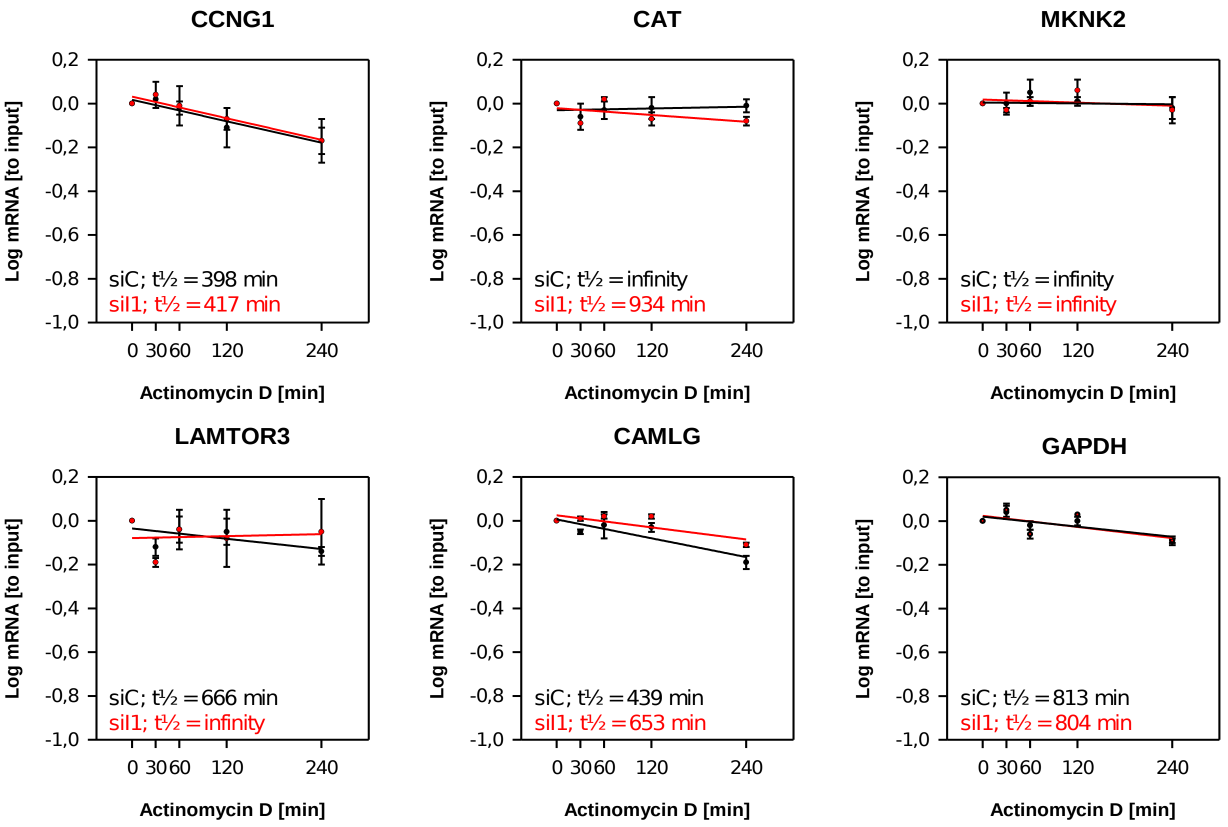

Figure S4. UNP$_{CLIP}$ mRNA decay upon Actinomycin D treatment. Decay was monitored by RT-q-PCR in control (black) and IGF2BP1-depleted (red) ES-2 cells upon indicated time of Actinomycin D treatment. Error bars indicate standard deviation. Average mRNA half-life, determined in three independent studies is indicated.

REFERENCES

Conway, A. E., Van Nostrand, E. L., Pratt, G. A., Aigner, S., Wilbert, M. L., Sundararaman, B., et al. (2016). Enhanced CLIP Uncovers IMP Protein-RNA Targets in Human Pluripotent Stem Cells Important for Cell Adhesion and Survival. *Cell Rep* 15, 666–679

Hafner, M., Landthaler, M., Burger, L., Khorshid, M., Hausser, J., Berninger, P., et al. (2010). Transcriptome-wide identification of RNA-binding protein and microRNA target sites by PAR-CLIP. *Cell* 141, 129–141
