# Supplementary figures and images for: IGF2BP1, a Conserved Regulator of RNA Turnover in Cancer
Source: Front Mol Biosci. 2021 Mar 22;8:632219. doi: 10.3389/fmolb.2021.632219 (PMC8019740; doi:10.3389/fmolb.2021.632219)

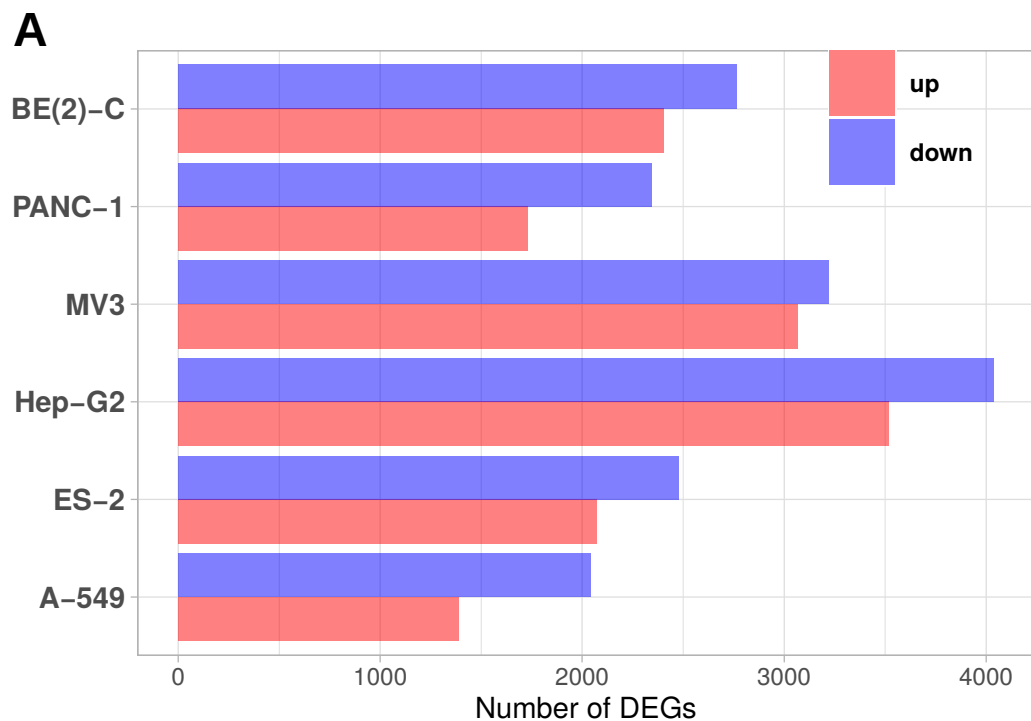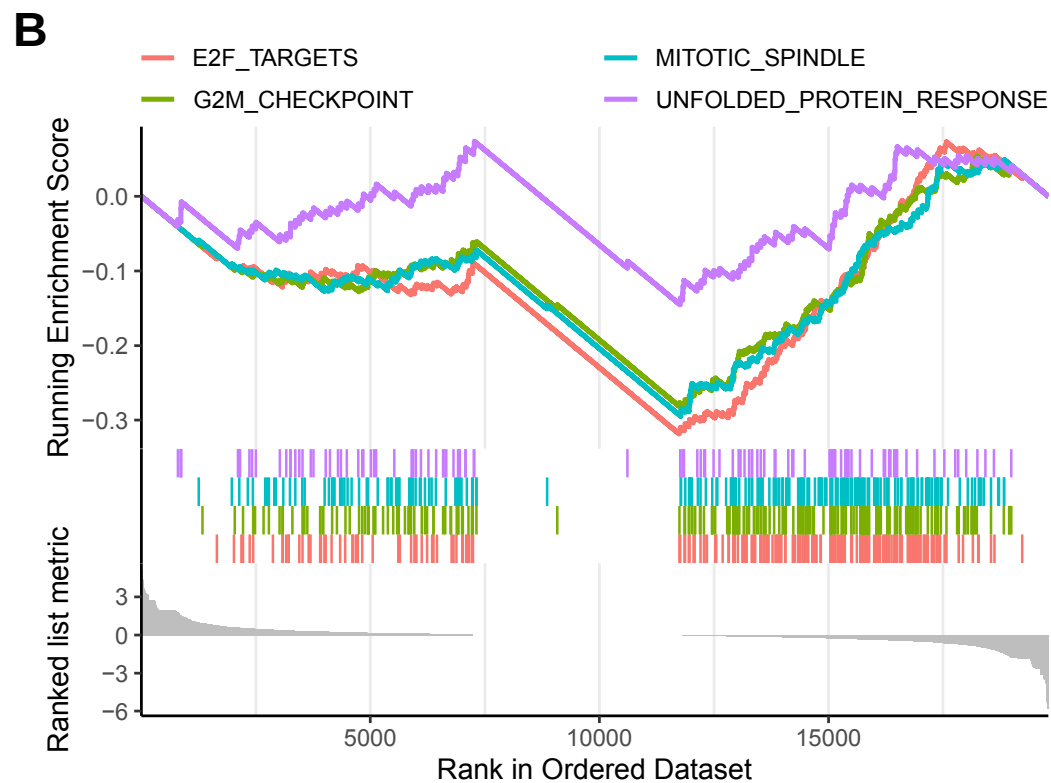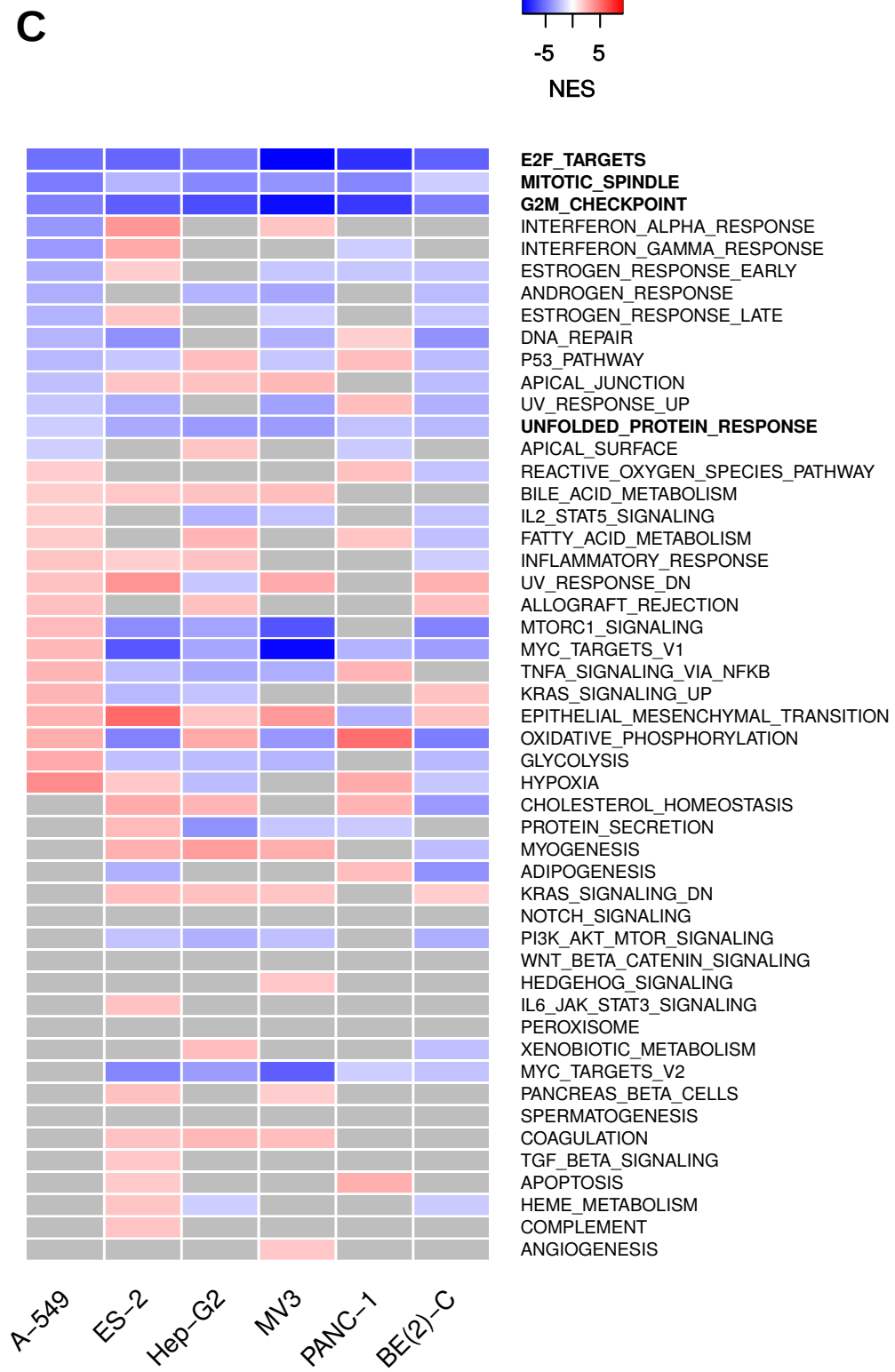

Supplement: Supplementary file 3 [file datasheet3.zip › figure1.pdf]

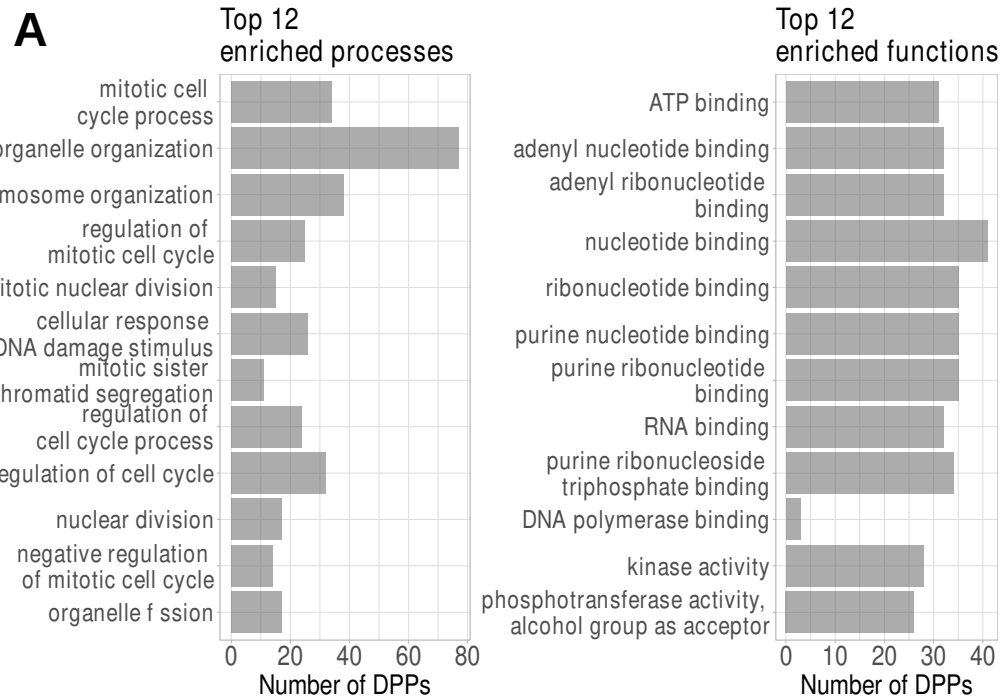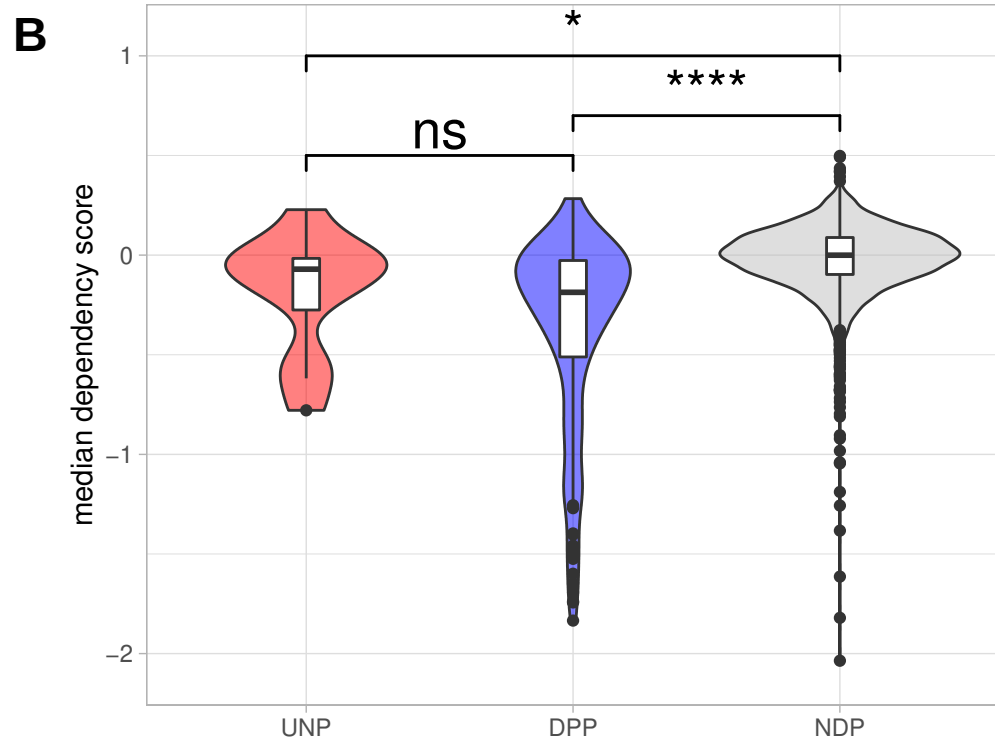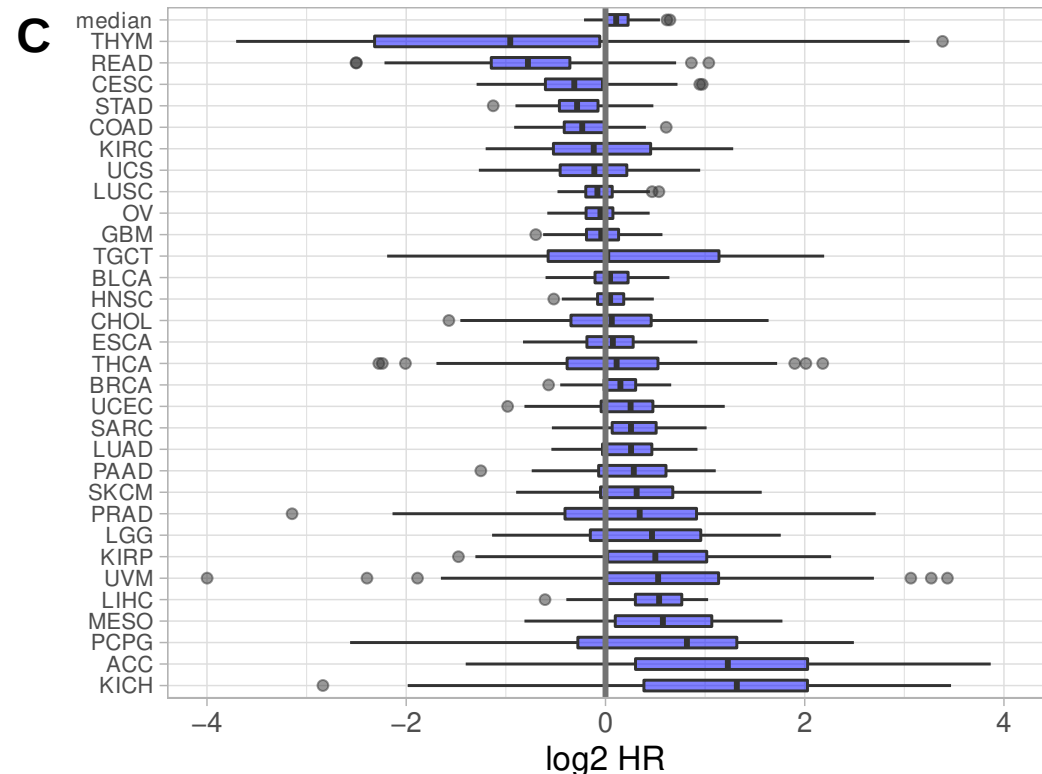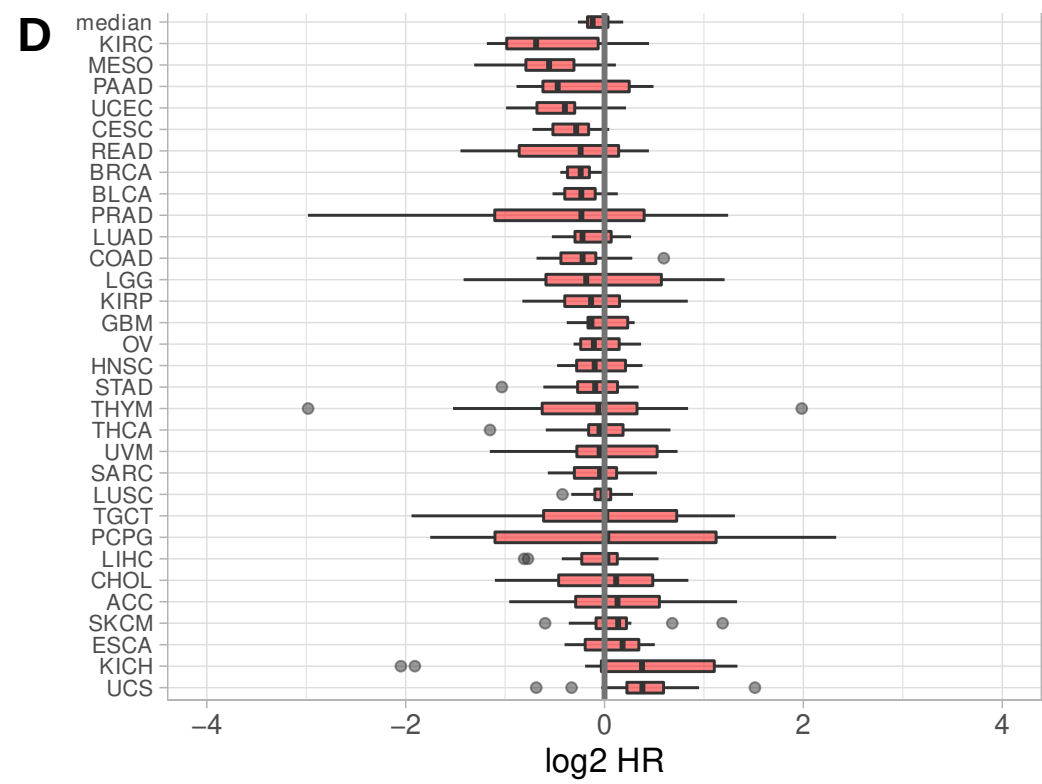

Supplement: Supplementary file 3 [file datasheet3.zip › figure2.pdf]

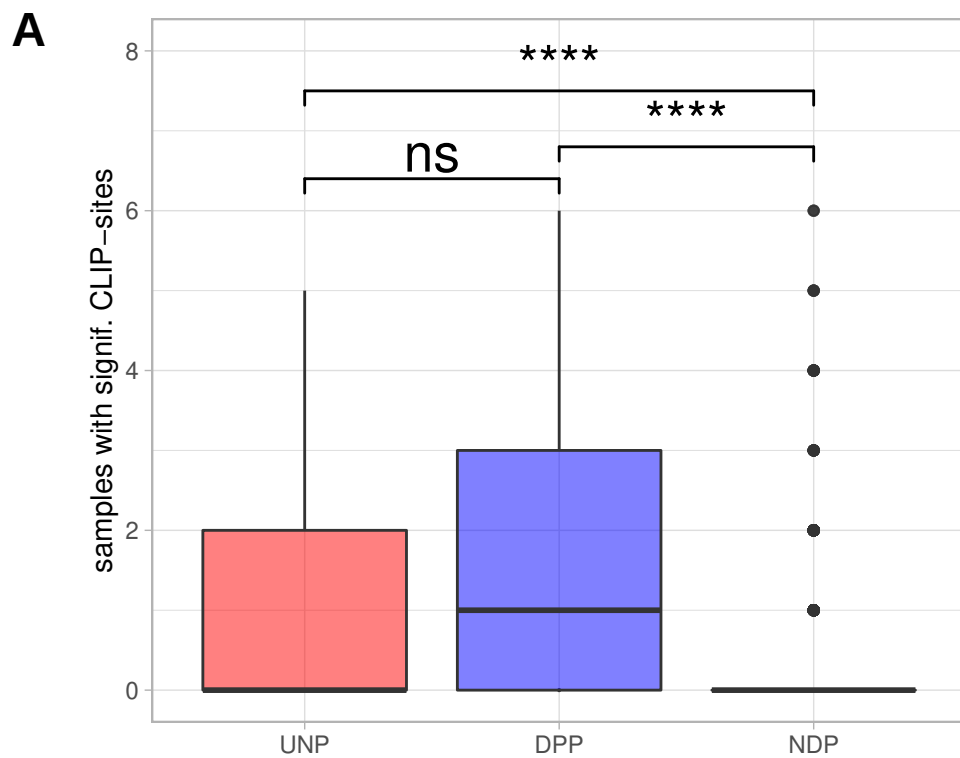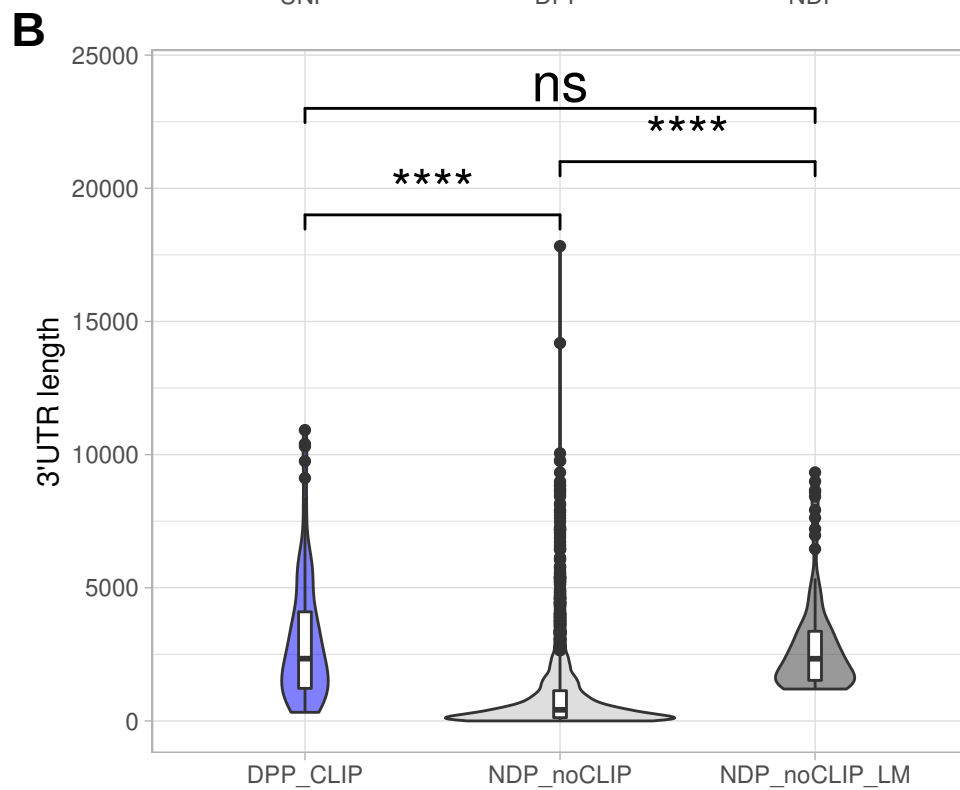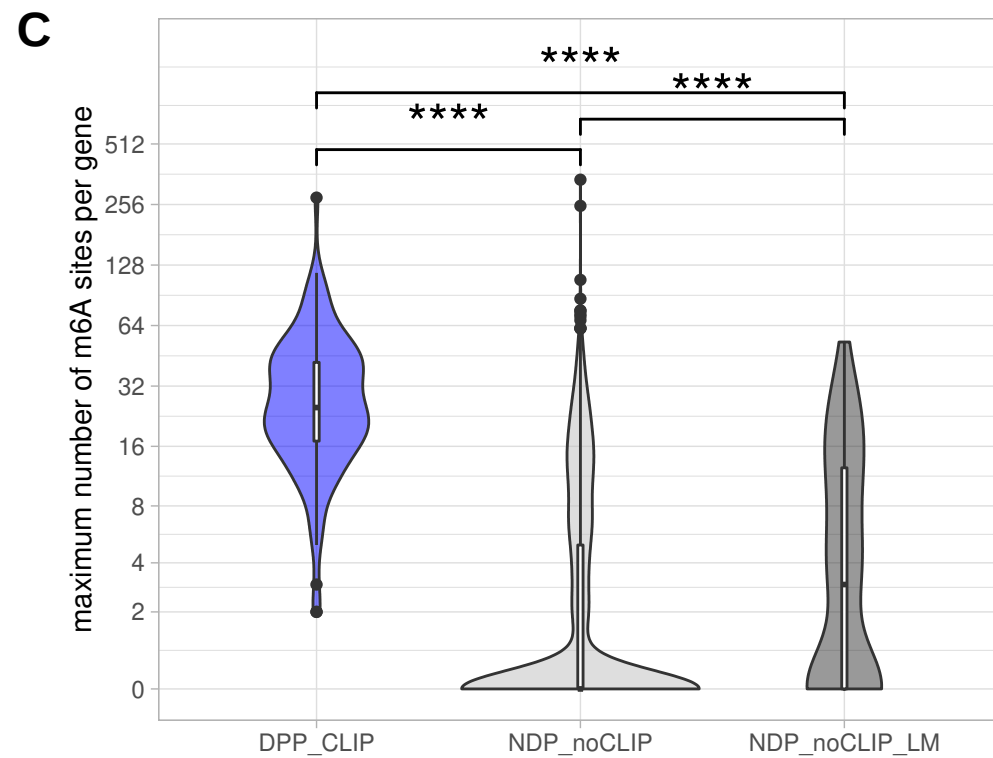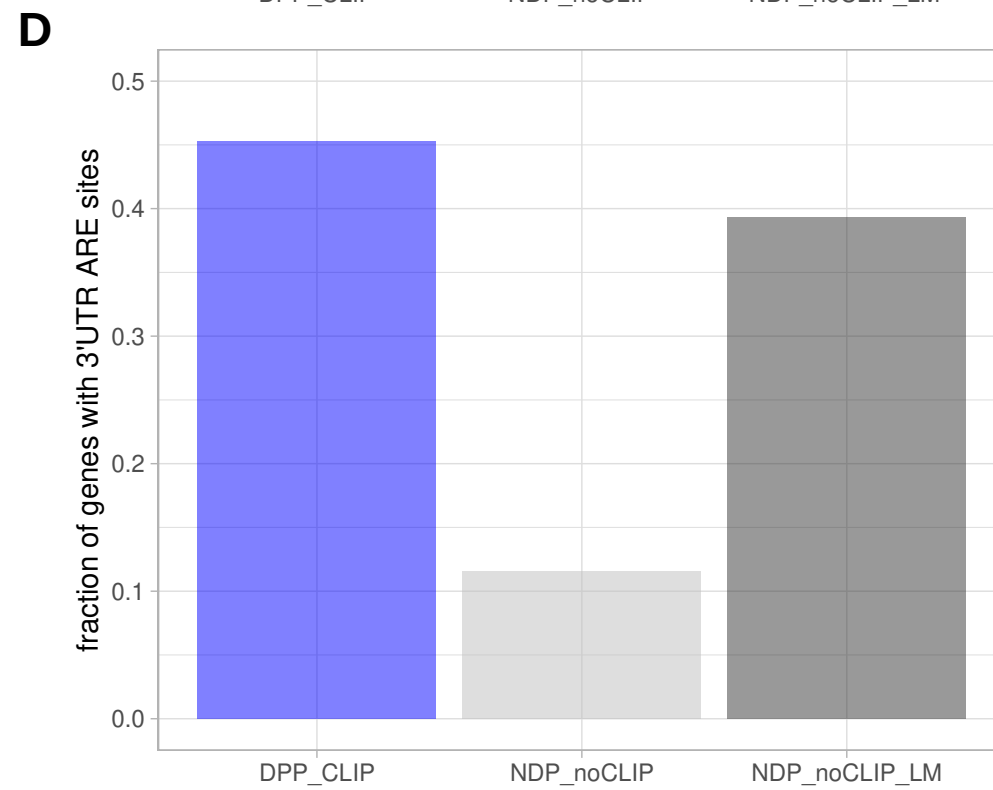

Supplement: Supplementary file 3 [file datasheet3.zip › figure3.pdf]

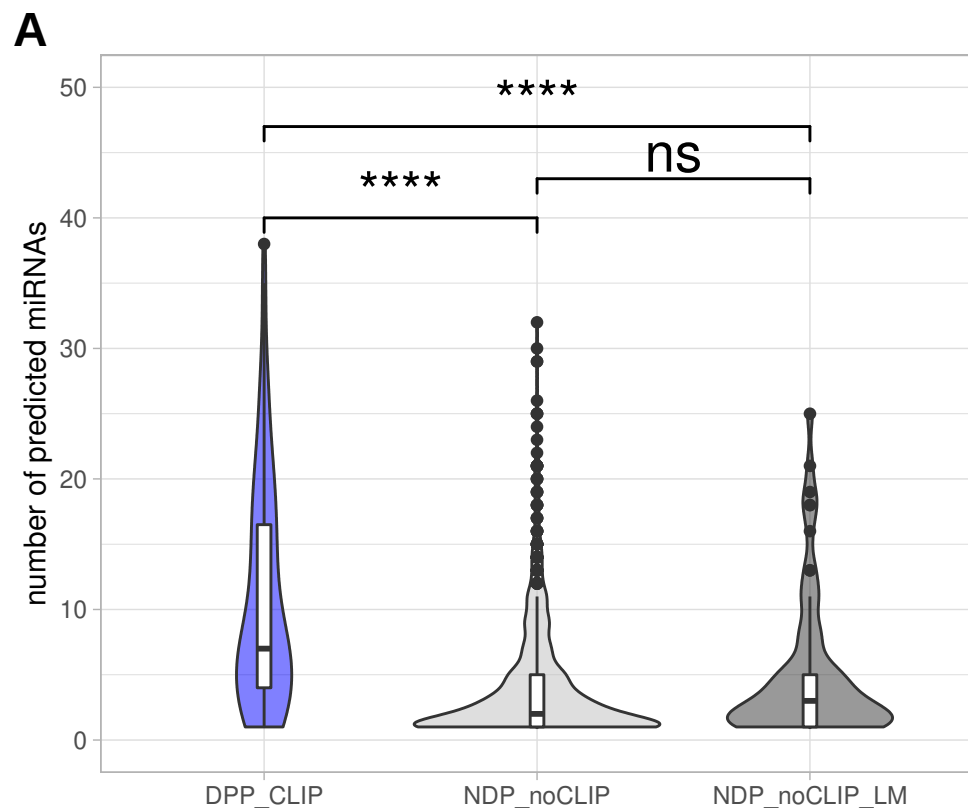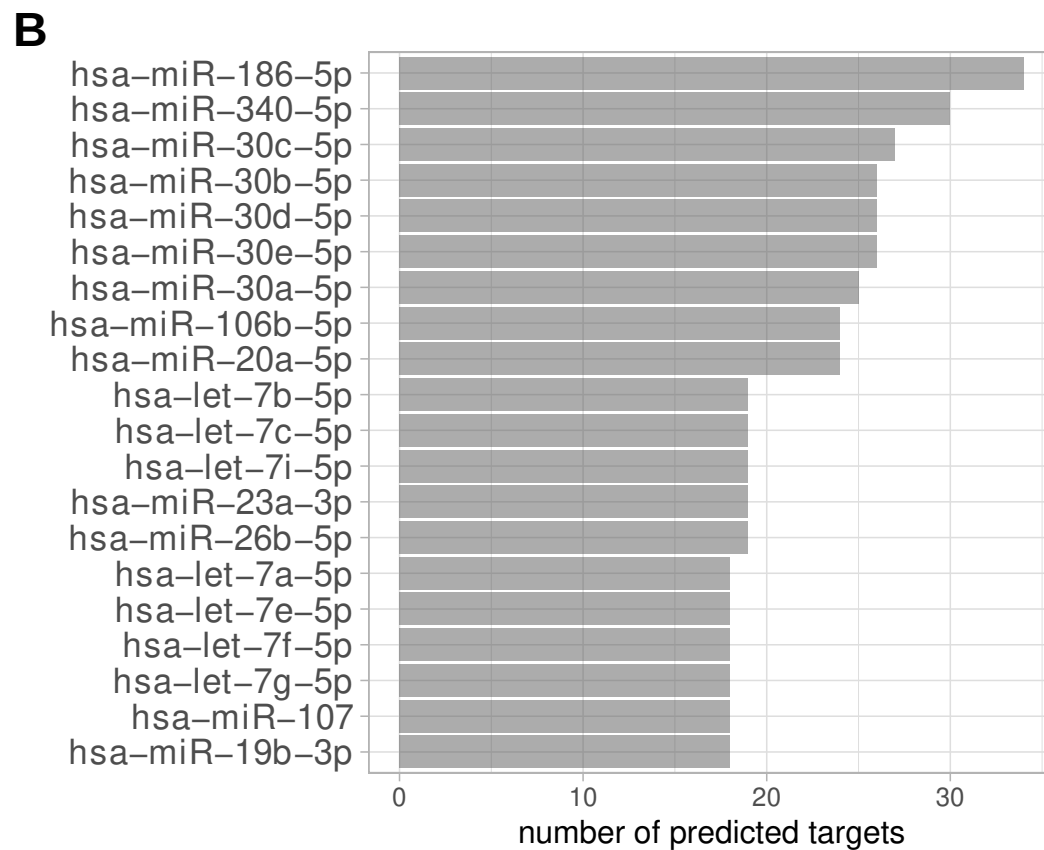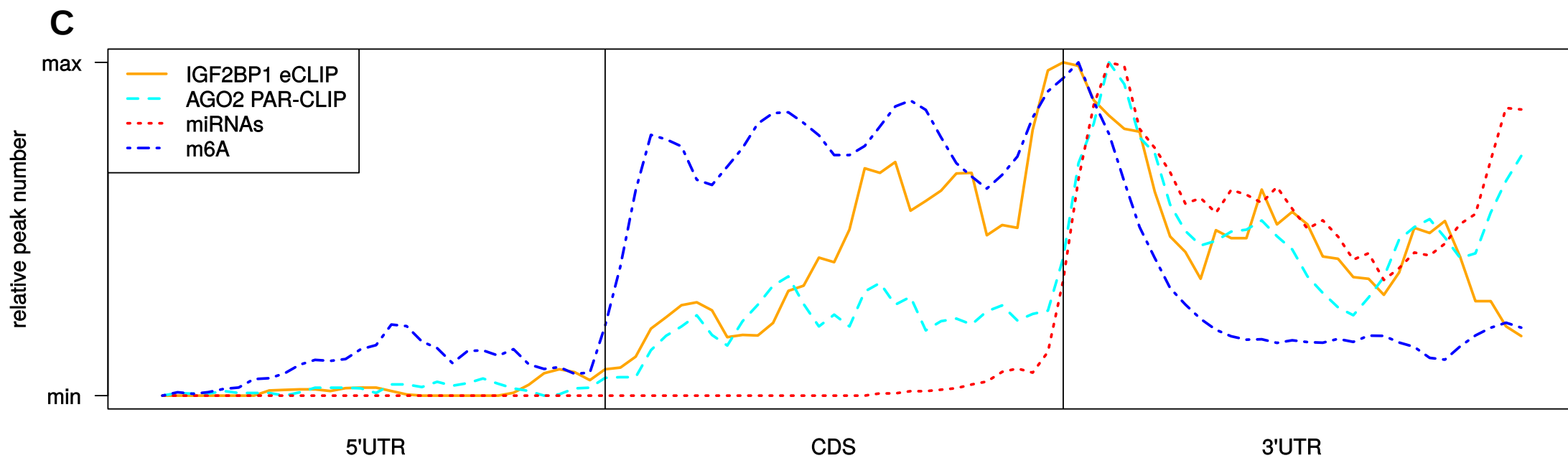

Supplement: Supplementary file 3 [file datasheet3.zip › figure4.pdf]

**A**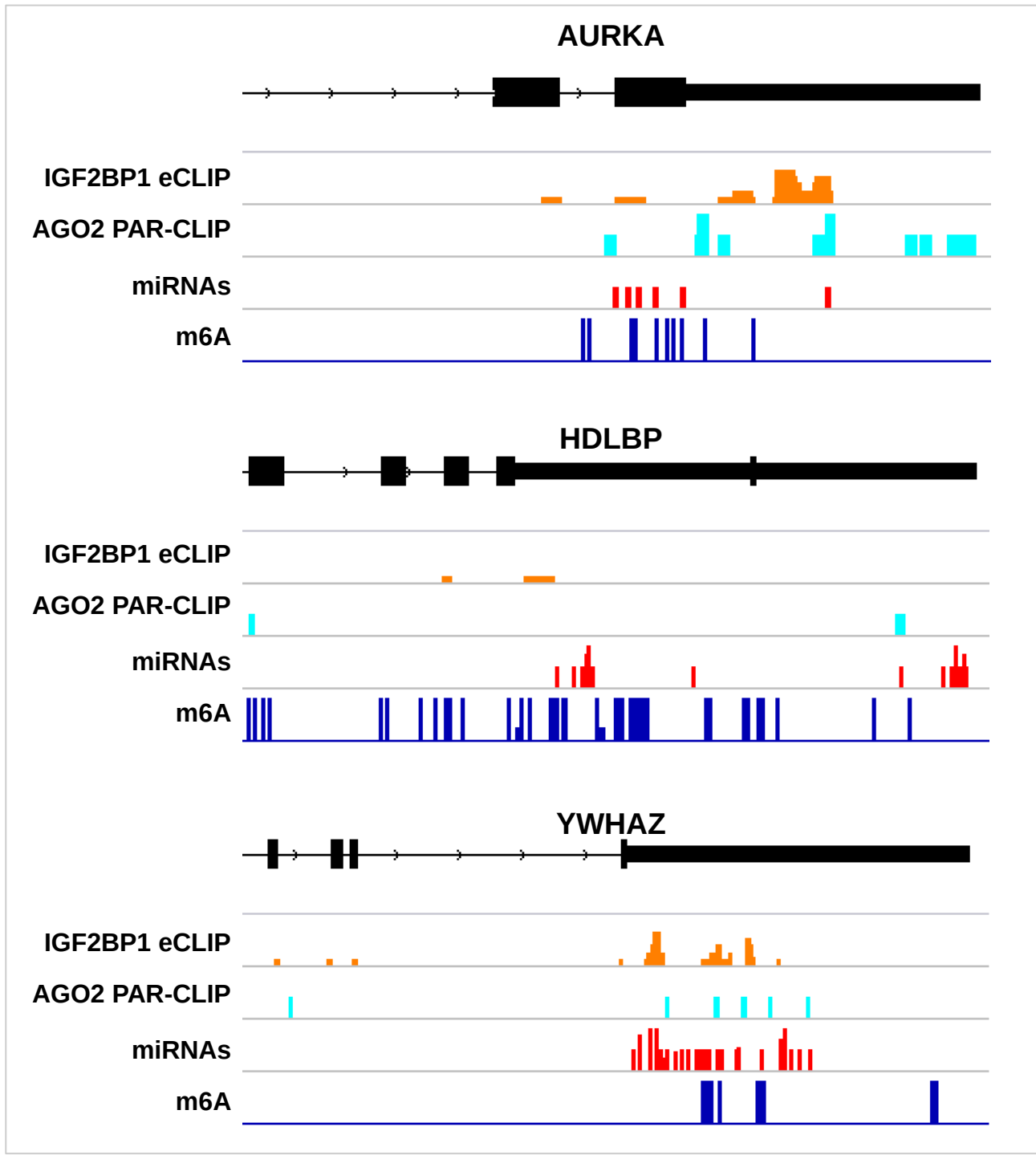**B**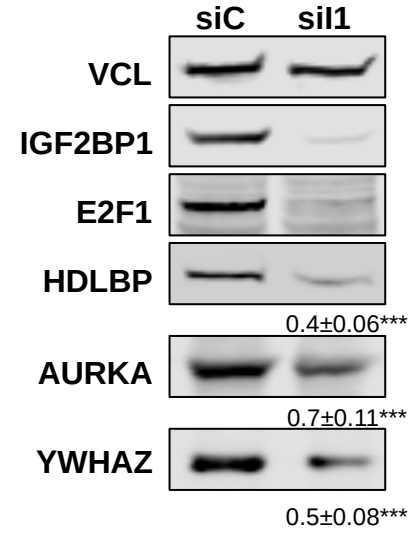**C**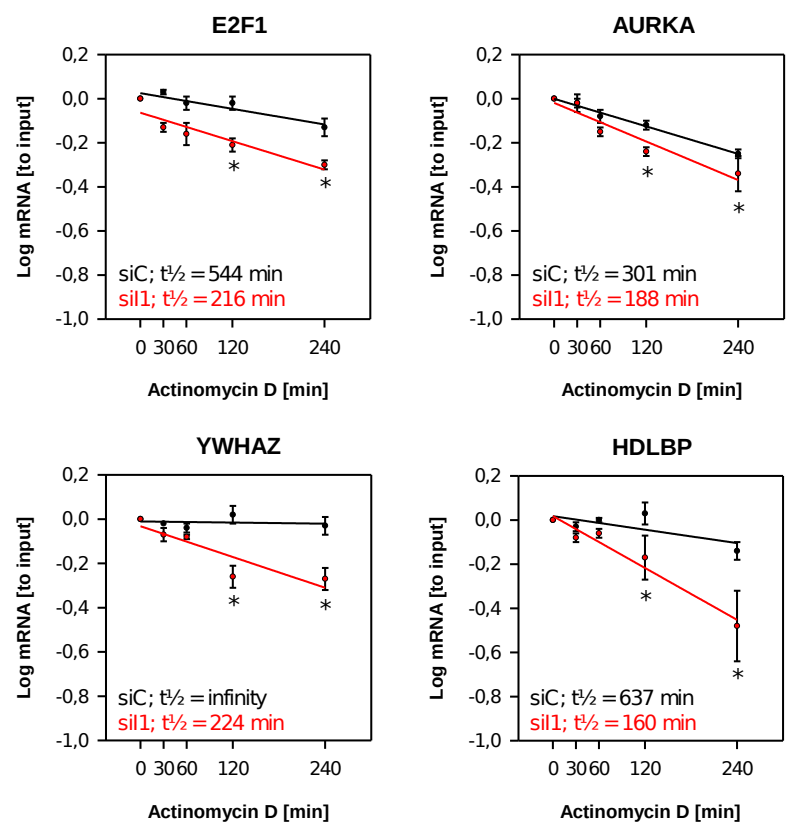

Supplement: Supplementary file 3 [file datasheet3.zip › figure5.pdf]
